# General practitioner utilization by age, sex and disease in Norway from 2012–2023: a national health registry study

**DOI:** 10.1080/02813432.2026.2666623

**Published:** 2026-05-11

**Authors:** Bjørn-Atle Reme, Per Minor Magnus, Siri Eldevik Håberg, Jonas Minet Kinge

**Affiliations:** a Department of Health Management and Health Economics, University of Oslo, Oslo, Norway; b Center for Fertility and Health, Norwegian Institute of Public Health, Oslo, Norway; c Department of Global Public Health and Primary Care, University of Bergen, Bergen, Norway; d Centre for Disease Burden, Norwegian Institute of Public Health, Oslo, Norway

**Keywords:** Health care demand, general practitioners, trends, mental health, population health

## Abstract

**Objective**: To describe trends in the number of general practitioner consultations in primary care, stratified by age, sex, and diagnosis.

**Method:**

Register-based population-wide repeated cross-sectional study. Data from the Norwegian Control and Distribution of Health Reimbursement (KUHR) database, linked to data from the Norwegian Population Registry.

**Participants:**

All Norwegians alive and living in the country each year from 2012 to 2023. The population grew from 4,985,870 in 2012 to 5,488,984 persons in 2023 during this period.

**Main outcome measure:**

The average annual number of general practitioner consultations per inhabitant in primary healthcare.

**Results:**

The average number of yearly General Practitioner (GP) visits per inhabitant increased from 3.1 to 3.6 for women and from 2.2 to 2.4 for men from 2012 to 2023. Age-specific utilization trends revealed the largest increase among 16–19-year-old women, whose GP visits rose from 2.05 to 2.99 per year. Diagnosis-specific trends highlighted a substantial increase in consultations for mental illness among women, from 0.31 to 0.46 per inhabitant, with the largest relative growth among 6–15-year-old girls (+139%). Conversely, consultations for heart disease saw the largest relative decline, particularly among women aged 67 to 79 (−68.3%) and men aged 80 to 89 (−0.94 consultations per inhabitant).

**Conclusions:**

Primary care utilization has increased over the past decade, with notable variation by age, sex, and diagnosis. The particularly strong increase in consultations for mental illness among young individuals highlight the potential for considering other medical professionals, such as specially trained nurses, to serve as the primary point of contact for young individuals with mental health challenges.

## Background

In Norway and many other countries, the general practitioner (GP) service is under pressure [1–5]. The pressure stems from a combination of several factors, including changes in both healthcare accessibility and demand. On accessibility, the number of GP lists has grown from 4440 to 5379 between 2012 and 2023, while the average length of GPs’ lists declined over the same period [6–8]. On demand, population growth and a rise in average yearly visits – from 2.6 to 3.0 per resident – have expanded service use over the same period [9]. The pressures have been particularly pronounced in rural areas, where recruitment challenges have left many patients without a regular GP [10]. While considerable attention has been given to addressing the challenges in the recruitment and retention of healthcare professionals, relatively little focus has been placed on understanding and managing the increasing demand for services.

For efficient planning, it is crucial with an accurate understanding of changes taking place on the demand side. Previous studies describing developments in primary care utilization and morbidity use limited population-based survey samples or focus on specific diseases [11–15]. However, studies covering the entire population – including utilization patterns by age, sex and calendar year – are lacking.

The aim of this study was to describe age-, sex- and diagnosis-specific counts and trends in general practitioner consultations in primary care.

## Methods

The study population included all Norwegians citizens residing in Norway in each of the years from 2012 to 2023. The data presented in this study were retrieved from Statistics Norway’s webpage, which provides access to statistics on primary health care utilization, including consultations with general practitioners, for a wide range of subgroups, including disease categories, age groups and gender [16]. For this study, emergency visits were excluded; hence, we report developments in regular visits to GPs.

In Norway, the GP service is usually the first contact for the population if ill or in need of health services. The service is free for children under the age of 16, and adults pay a user fee of 15 to 50 EURO, which counts toward an exemption card after the patient has paid the maximum limit of about 320 EURO [17]. For the general practitioner to be reimbursed, all consultations, with diagnoses and reimbursement codes are registered in the Norwegian Control and Payment of Health Reimbursements Database (KUHR), according to the International Classification of Primary Care (ICPC-2) [18]. In ICPC-2, codes 01–29 denote symptoms/complaints, whereas codes 70–99 denote disorders/diagnoses. Because coding often reflects the patient’s reason for encounter rather than a confirmed diagnosis, we refer to these categories as ‘disease-type’ for brevity, acknowledging that some entries reflect symptoms. See the Supplementary materials for more information about which reimbursement codes were the basis of counted consultations in this study.

The subcategorization of ICPC-2 used in this study is based on a collaborative effort between Statistics Norway and medical experts, either due to similar etiology, approach, or treatment. The groups represent some of the most common reasons for patients visiting their GP. See Supplementary information for detailed information on which diagnoses belonged to each grouping.

### Statistical analysis

The design is cross-sectional, repeated in each calendar year. We are not following individuals over time. The same individual may occur several times in each yearly measure. We estimated sex-, age- and disease-specific average number of GP-consultations per inhabitant each year from 2012 to 2023 in three steps: (i) sex- and age group-specific estimates, (ii) sex- and disease-type specific estimates, and (iii) the intersection of sex-, age- and disease-specific estimates. In (i) and (ii), we report the change in the average number of consultations per inhabitant, while in (iii) we report both percent change and absolute changes. Using percentage changes in this step allowed us to include broader subgroups, such as ‘all ages’ and ‘all diagnoses’, without introducing issues of scale or comparability.

The aggregate data available from Statistics Norway does not include variance estimates of the averages, which limits the possibility for inference. To allow inference, we used an individual-level dataset of primary care utilization from 2019 (access based Regional Committee for Medical and Health Research Ethics South-East Norway approval 2018/434). This dataset was linked to the population registry to impute variance for the conditional means reported in this study. Using the variation in means within this dataset for the subgroups assessed in this study, we constructed a simple regression model allowing us to impute variance in this dataset. Generally, these models had a very high level of fit, all with $R^{2}$>0.9, implying that our modelling of variance with regression models were quite efficient. Detailed documentation of the methodology and imputation process is provided in the Supplementary Information.

Given the large population size in this study, the standard errors are small, and not easily discernible in figures. As a result, we chose not to include standard errors in all figures. However, confidence intervals, along with tests of differences in means, are provided in the corresponding tables in the Supplementary Information.

## Results

**Figure 1.**
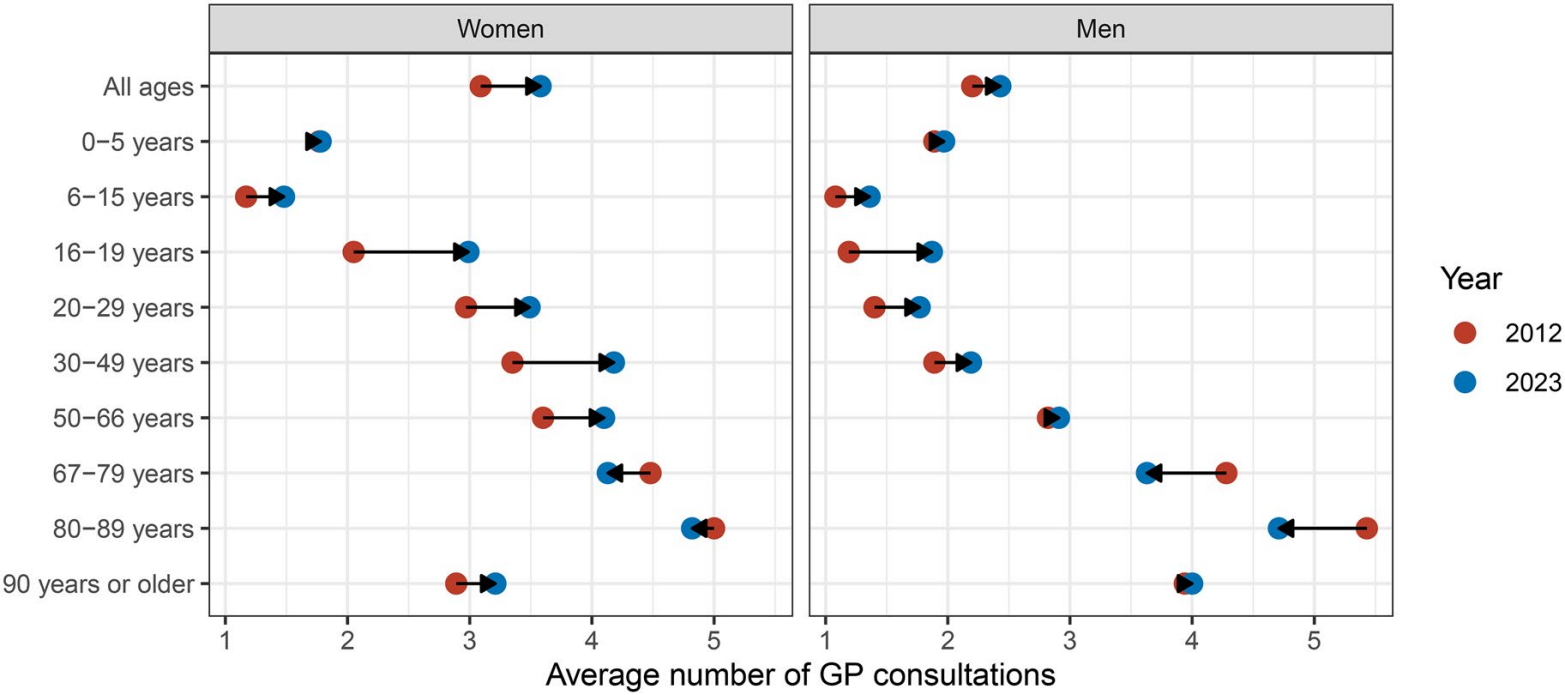
Average number of GP consultations per inhabitant for different age-groups, by sex.

The Norwegian population increased from 4,985,870 in 2012 to 5,488,984 in 2023. The average number of GP visits per inhabitant per year rose from 3.1 to 3.6 for women and from 2.2 to 2.4 for men over the same period. Age-specific analysis revealed increased primary care utilization among younger age groups, contrasted by a general decline among individuals aged 67 and older (Figure 1). The largest increase was observed among 16–19-year-old women, from 2.05 to 2.99. Conversely, the largest decrease was seen among 80–89-year-old men, with consultations declining from 5.43 to 4.71. See Supplementary Table 1 for a corresponding table and Supplementary Figure 1 for a by-year development.

**Figure 2.**
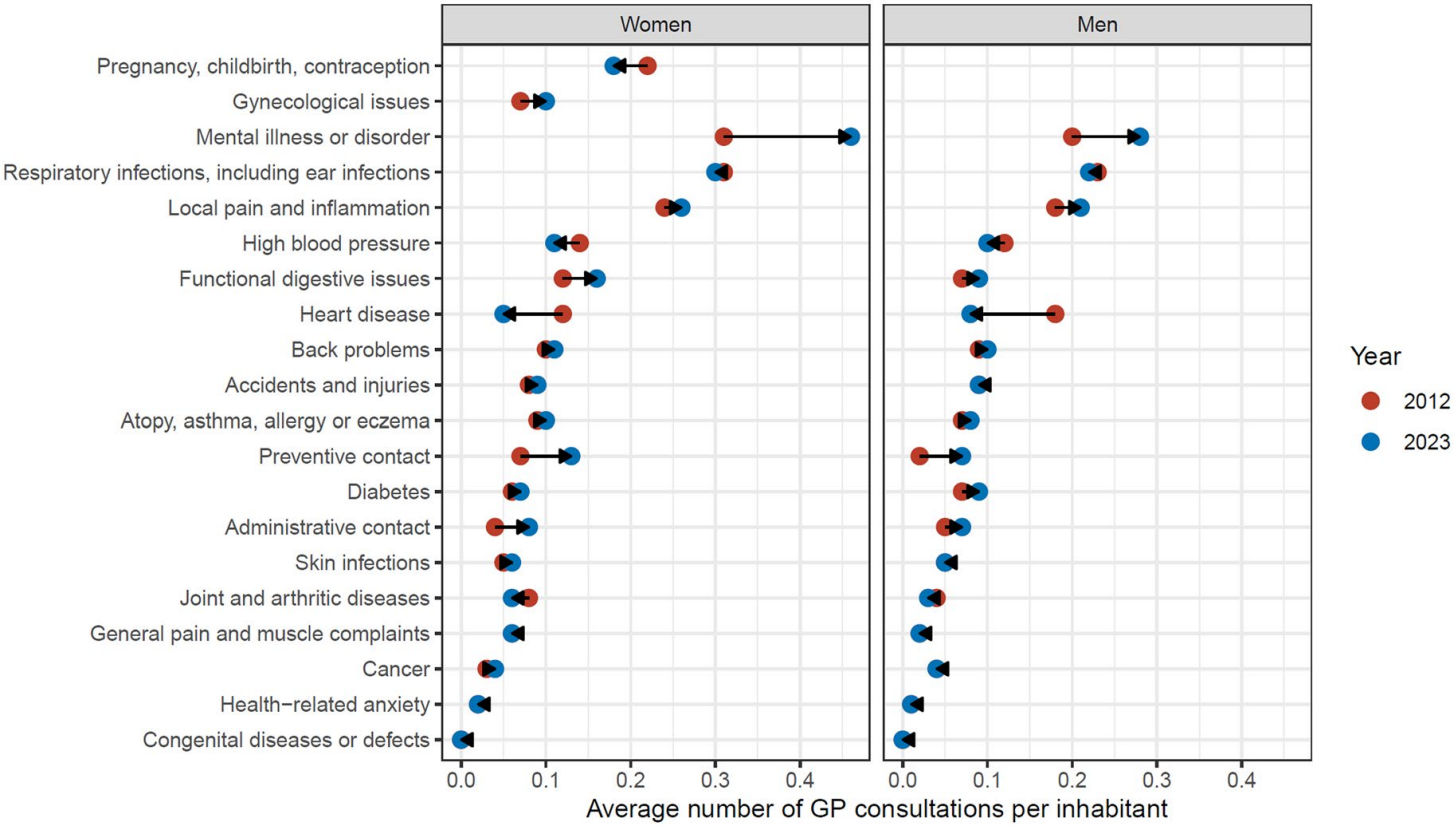
Average number of GP consultations per inhabitant for different disease-groups, by sex.

Regarding disease-specific trends, the largest increase was observed for consultations related to mental illness among women (Figure 2), with an increase from 0.31 to 0.46. The largest decrease was related to heart disease among men, from 0.18 to 0.08. See Supplementary Table 2 for a corresponding table and Supplementary Figure 2 for a by-year development.

When combining age- and disease-specific data, the largest increase, measured in percent, was found for mental illness among young women (Figure 3A). Specifically, the greatest relative increase was found for mental illness among girls aged 6 to 15 (+139%), while the largest reduction was observed for heart disease among women aged 67 to 79 (−68%). Notably, the sharp rise in respiratory health consultations among 16–19-year-olds is likely linked to the high school absence reform since 2016, which introduced stricter documentation requirements for attendance. See Online Supplementary Material for age, sex and disease type-specific development for each year.

Figure 3. (A) Percent change in the number of GP consultations from 2012 to 2023 across age-group and disease-type, by sex. We only included combinations of age-group and disease type where the highest rate per 1000 inhabitants was more than 0.01 consultations, to avoid high sensitivity to low base rates. (B) Change in the average number of GP consultations from 2012 to 2023 across age-group and disease-type, by sex. Stars show significance of the difference, *** is *p* < 0.001; ** is *p* < 0.01, * is *p* < 0.05.

**Figure:**
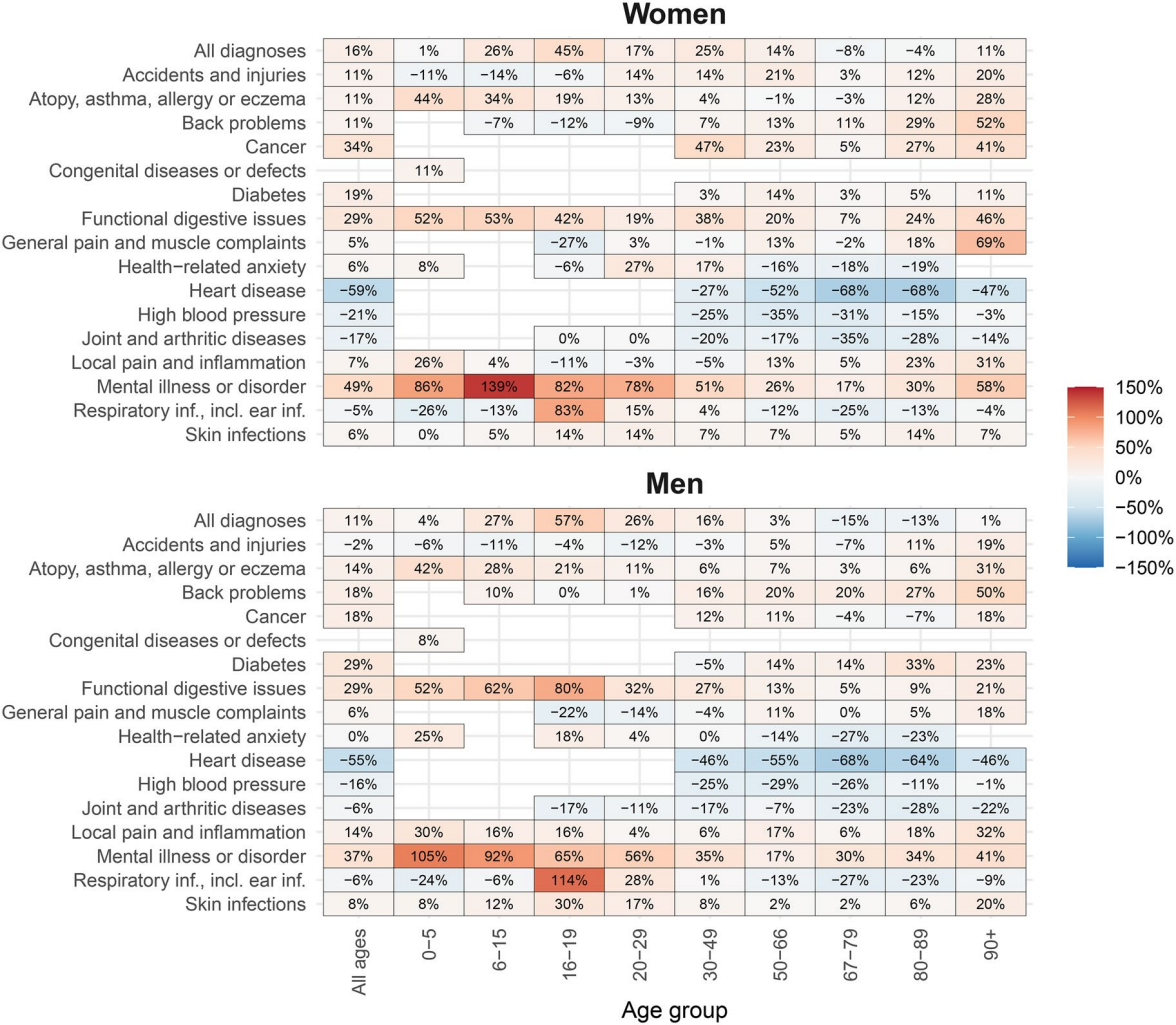

**Figure:**
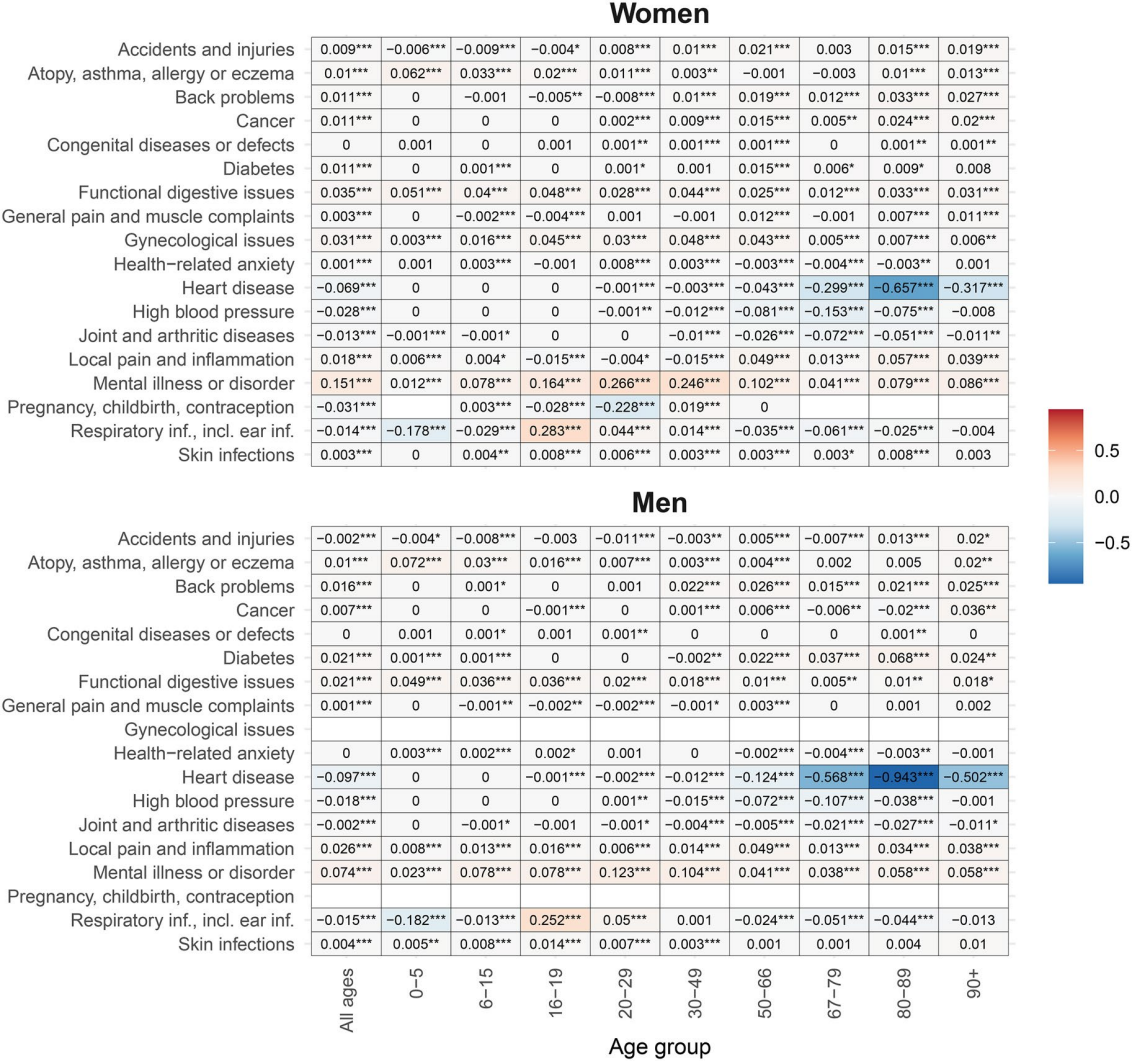

The strongest change in absolute terms was found for heart disease among men aged 80 to 89, corresponding to −0.94 consultations per inhabitant (Figure 3B). The largest increases were found for respiratory problems and mental illness among young women.

## Discussion

The average number of general practitioner consultations increased from 2012 to 2023 in Norway. Age-specific utilization trends revealed the largest increase among 16–19-year-old women, while utilization in individuals above age 67 decreased. Diagnosis-specific trends highlighted a substantial increase in consultations for mental illness among women, with the largest relative growth among 6–15-year-old girls. Consultations for heart disease had the largest relative decline, particularly among women aged 67–79 and men aged 80–89.

The increased utilization of health services might be caused by an increase in the prevalence of diseases. According to the Global Burden of Disease studies, mental disorders rank among the leading causes of disability [19]. Depressive and anxiety disorders, for example, ranked second and eighth in 2019 (YLD – years lived with disability) [20,21]. While an important cause of disability in all age groups, mental disorders have consistently been an even larger source of disability in the lower age groups. Our findings are in line with previous studies from both Norway and other countries highlighting an increase in mental health issues among adolescents and young adults [11,22–25]. Moreover, several of these studies find that although this growth is present for both sexes, it is particularly strong among 6–15-year-old girls. These patterns also align with a recent comprehensive study of Norwegian health expenditures showing that mental disorders are the primary cost driver among younger cohorts [26].

The decline in healthcare utilization among the elderly, particularly related to heart-related problems, mirrors the falling rate of heart-related disease reported in other studies, both from Norway and internationally [27–29]. These changes are also consistent with recent evidence of increased use of statins [30].

The changes observed in this study, particularly related to mental illness, not only reflect changes in morbidity but also changes in the threshold for seeking professional help when struggling emotionally. This phenomenon has often been referred to as ‘medicalization’, where experiences previously considered as a normal part of life to a larger extent are considered as disease with need for professional treatment [31]. Moreover, these developments could to some extent reflect increasing availability of treatment for conditions that could previously not have been efficiently treated.

The sharp rise in respiratory consultations among 16–19-year-olds likely reflects the high school absence reform introduced in August 2016, which required medical documentation for absences exceeding 10% in a subject, rather than a true increase in respiratory morbidity. This illustrates how administrative incentives may shape healthcare utilization patterns.

Compared to other countries, the average levels of healthcare utilization reported in this study – 3 consultations per year – is relatively modest, as a recent OECD report found an average of six primary care doctor consultations per person per year [32]. At the same time, the report finds considerable variation depending on payment scheme, and the levels for Norway are comparable to those found in Denmark, Sweden and Finland.

Importantly, the overall increase in healthcare utilization, particularly for younger individuals, highlights that pressures on primary care provision likely will continue to grow as the younger individuals age. This shift in demand indicates a need to expand capacity for mental-health care over the coming decades. Many of the mental health concerns currently managed by general practitioners could potentially also be addressed by specially trained nurses or other health professionals certified in cognitive behavioral therapy, as demonstrated in pilot programs in the UK [33–35].

### Strengths and limitations

The main strength of this study is that it covers the full population across a wide range of disease types and age groups, hence providing a detailed picture of the overall trends in primary care utilization. The study also has limitations. First, aggregate data limits the possibility of studying fine-grained groups, such as particular sub-groups of mental illnesses or smaller age groups. This also limits the extent to which the study can account for population-level social shifts, changes in health-care organization and policy, hence, confounding factors may exist. Second, as Statistics Norway only reports means, the inference is based on extrapolation for the variability of the data from one particular year. To the extent that the data-generating mechanism is fairly consistent over time – in particular the relationship between means and variances – this method still provides a credible estimate of inference. Moreover, the study reports from the full population, which makes inference in itself less relevant, as the sample is the full population. This also naturally must result in very small standard errors, and that almost all changes are statistically significant using standard statistical test. Third, in primary care, physicians are only required to submit one ICPC2 code to be reimbursed. Hence, although the primary diagnosis has been found to correspond well with patient records, comorbidities are likely underreported in the GP data and important information about the reason for encounter could be missing [36,37]. If some health conditions are thought of more often as secondary diseases, and not reported by the GP, the utilization for these conditions will be underestimated. Fourth, a diagnosis is the key to various social benefits in Norway and many of the disorders are essentially subjective. Hence, despite having the same symptoms, patients may be labelled differently by different physicians, and differently across countries depending on the social security system [38]. Last, the rationale underlying the ICPC-2 subcategorization chosen by Statistics Norway has not, to our knowledge, been clearly documented.

## Conclusions

This study documents a significant increase in primary care utilization in Norway from 2012 to 2023, particularly among younger individuals due to mental health issues, while consultations for heart disease among older individuals have declined. Given the growing prevalence of mental-health problems, our findings suggest exploring task-shifting so that non-GP clinicians provide a greater share of care for mild-to-moderate conditions.

## Supplementary Material

Supplementary Material.docx

Online Supplementary.pdf

## Data Availability

The code to reproduce the study is available from the corresponding author upon request.

## Generative artificial intelligence (AI) statement

ChatGPT (version 5) was used to assist with language editing, grammar improvement and R-coding in the preparation of this manuscript.

## Data availability statement

The data used in this study, except for data used to estimate confidence intervals, was downloaded from Statistics Norway, and is available here: https://www.ssb.no/en/helse/helsetjenester/statistikk/allmennlegetjenesten
