# Supplementary material for: General practitioner utilization by age, sex and disease in Norway from 2012–2023: a national health registry study
Source: Scand J Prim Health Care. 2026 May 11;44(1):2666623. doi: 10.1080/02813432.2026.2666623 (PMC13162542; doi:10.1080/02813432.2026.2666623)

**Supplementary information**

This document serves as supplementary material for the study titled "General Practitioner Utilization by Age, Sex, and Disease in Norway from 2012-2023: A National Health Registry Study.", by Reme, Magnus, Håberg and Kinge.

**Table of contents**

Explanation of consultation data and disease categorization . . . 2-3

Estimations underlying imputed variance. . . . . . 4-6

**Supplementary Table 1**. Average number of GP consultations in primary care per

inhabitant for different age groups, by sex. . . . . . 7

**Supplementary figure 1**. Average number of GP consultations in primary care per

inhabitant for different age groups, by year and sex. . . . . 8

**Supplementary table 2**. Average number of GP consultations in primary care per

inhabitant for different age groups, by sex. . . . . . 9

**Supplementary Figure 2**. Average number of GP consultations in primary care per

inhabitant for different age groups and year, by sex. . . . . 10

**Consultation data and disease categorization**

**Consultation data:** The following reimbursement codes are included in Statistic Norway’s measurement: 2ad (consultation with a general practitioner, daytime rate), 2ak (consultation with a general practitioner, evening rate), 2ed (group treatment), and 2ae electronic consultation (new as of July 1, 2013). Other interactions, such as "simple patient contacts" (including simple inquiries and advice through personal attendance, letters, or phone calls, writing prescriptions, and sick notes without consultation), sample taking without consultation, and home visits, were excluded.

**Disease categorization:** These are assigned in connection with a consultation at a general practitioner (GP) or emergency clinic and are categorized into groups or chapters in the statistical database. The table of diagnostic groups is an aggregation of ICPC-2 codes into large groups that naturally belong together in general practice, either due to similar etiology, approach, or treatment. These groups represent some of the most common reasons for patients visiting their GP or the emergency clinic. The ICPC-2 codes included in each group are listed below:

- **Respiratory infections, including ear infections**: R05, R09-R23, R71-R83, H71-H74
- **Localized pain, inflammation, and other issues in the neck, shoulders, arms, and legs**: L01, L08-L17, L83, L87, L92, L93
- **Back problems**: L02-L03, L84-L86
- **General pain and muscle issues**: A01, L18-L19, L29
- **Joint and rheumatic diseases**: L07, L20, L88-L91, L94
- **Mental illness or disorders**: P01-P26, P28, P29, P70-P99
- **Atopy/asthma/allergy/eczema**: F71, R02-R03, R07, R96-R97, A92, S02, S87-S88, S98
- **High blood pressure**: K85-K87
- **Heart disease – failure, angina, infarction, arrhythmia, and other conditions**: K74-K84
- **Diabetes**: T89, T90
- **Cancer**: A79, B72-B74, D74-D77, F74, H75, K72, L71, N74, R84-R85, S77, T71, T73, U75-U77, W72, X75-X77, Y77-Y78
- **Gynecological issues**: X01-X22, X28, X29, X70-X74, X78-X81, X84-X99
- **Functional digestive issues**: D1-D12, D17-D21, D84-D87, D90, D92, D93
- **Skin infections**: S03, S09-S11, S70-S76, S84-S85, S92-S93, S95
- **Accidents and injuries**: A80-A82, A84, A86, A88, B76-B77, D79-D80, F75-F79, H76-H79, L72-L81, L96, N79-N81, R87-R88, S12-S19, S80, U80, X82, Y80
- **Congenital diseases or defects**: A90, B78-B79, D81, F81, H80, K73, L82, N85, R89, S83, T78, T80, U85, X83, Y82-Y84
- **Pregnancy, childbirth, contraception**: W01-W99
- **Health-related anxiety**: A25-A27, B25-B27, D26-D27, F27, H27, K24-K27, L26-L27, N26-N27, P27, R26-R27, S26-S27, T26-T27, U26-U27, X23-X27, Y24-Y27
- **Administrative contact**: A97
- **Preventive contact**: A98, A981
- **Other diagnoses**: All other codes

It is also possible to sort diagnoses according to the chapter structure of the ICPC-2 coding system, which is the International Classification for Primary Care (developed by the Wonca International Classification Committee). This system allows for the registration of reasons for contact, health problems, and diagnoses. It emphasizes the location of symptoms or illnesses in the body, such as the eyes, ears, skin, etc. This means that cancer, for example, will be distributed across several chapters. There are 17 chapters in total in the classification system, based on general conditions (Chapter A), organ systems (14 chapters in total), mental disorders (Chapter P), and social problems (Chapter Z). The tables in the statistical database include only the primary diagnosis; secondary diagnoses are not included.

**Estimations underlying imputed variance**

The dataset used for modeling variance comprised a complete individual-level record of general practitioner (GP) consultations from 2019. Each entry represented a GP consultation linked to a diagnosis code. This dataset was further linked to the population registry to aggregate, for each type of disease, the number of consultations each inhabitant had for different diagnoses. To align this data with the age groups and disease categorizations used in the present study, we calculated the mean and variance of the number of consultations. This was done across three different models, corresponding to the analytical framework presented in this paper.

**General modelling strategy:** Since we had means available from Statistics Norway for years 2012 to 2023, the relationship between means and variances in the individual-level dataset was modeled as follows: First, we estimated group means and variances in the 2019 individual-level dataset. Second, using these means and variances, we estimated the following regression model to capture the relationship between means and variances in this type of data: $Variance=\beta_{1}\mathrm{Mean}+\beta_{2}{Mean}^{2}$, with no constant term. The data used for this modeling varied depending on the specific part of the analysis. Specifically, age-groups by sex, disease-groups by sex, and age- and disease-groups by sex. Generally, these models had a very high level of fit, all with $R^{2}$>0.9, implying that our modelling of variance with this quadratic regression model was quite efficient.

**Age-groups, all diseases, by sex (Supplementary Table 1):** We estimated the mean and variance for number of consultations by age-group and sex in the 2019 individual level dataset (corresponding to Figure 1 and Supplementary Table 1). This data was used as input in the regression model (see above). The result, $\beta_{1}=3.4$ and $\beta_{2}=0.91$ ($R^{2}=0.98)$, together with the actual reported mean from the Statistics Norway (${Mean}_{t})$ was used to estimate the variance in period t ($\hat{{Var}_{t}})$. Then, using the number of observations from the population register in the relevant year ($N_{t}$), we constructed the standard errors, as follows: ${SE}_{t}=\sqrt{\frac{\hat{{Var}_{t}}}{N_{t}}}$.

Below is a graphical presentation of the model fit:


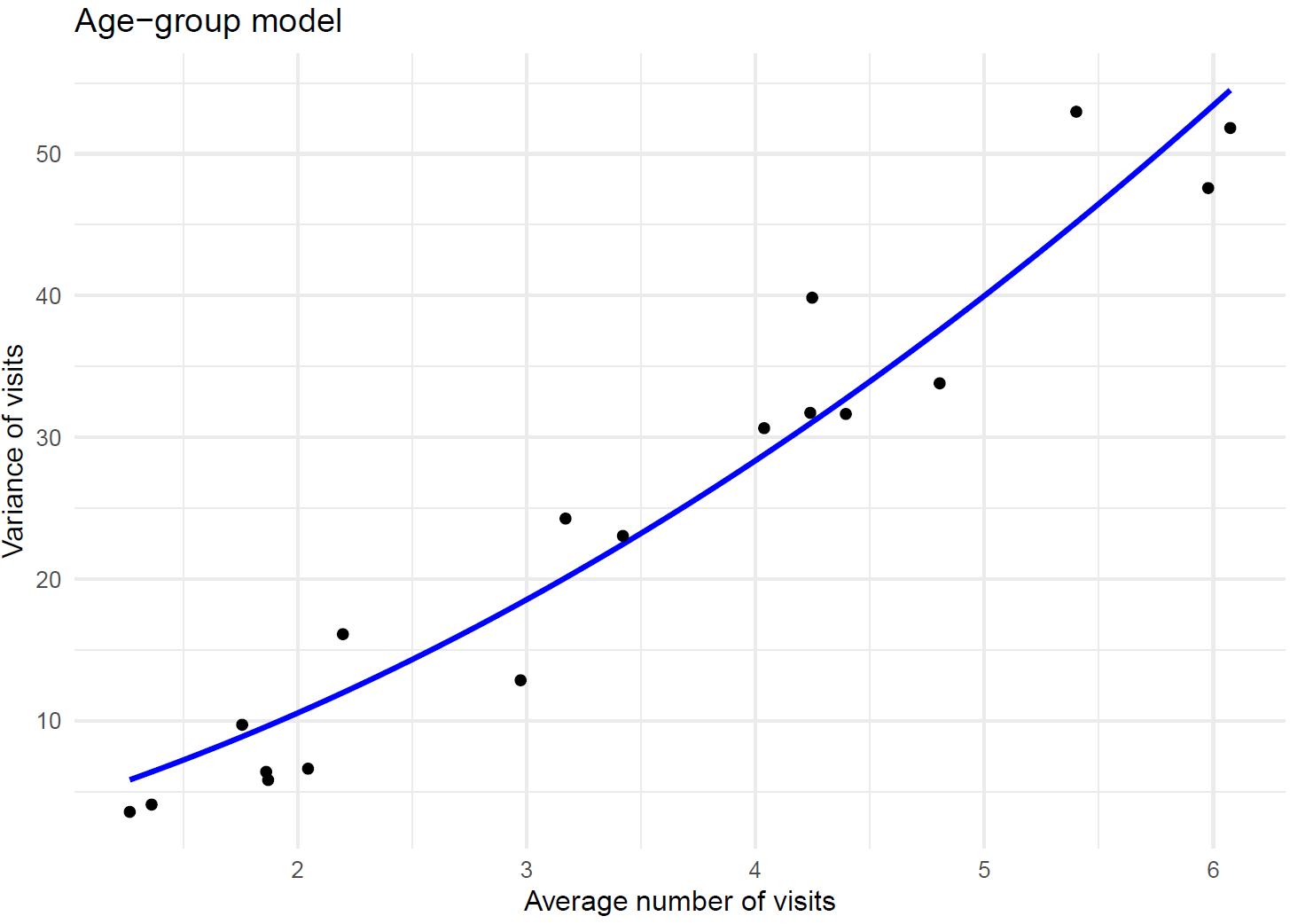


**Disease types, all ages, by sex (Supplementary Table 2):** Same procedure as described above, but grouping by disease types instead of age groups. the result $\beta_{1}=4.65$ and $\beta_{2}=0.001$ ($R^{2}=0.933)$.


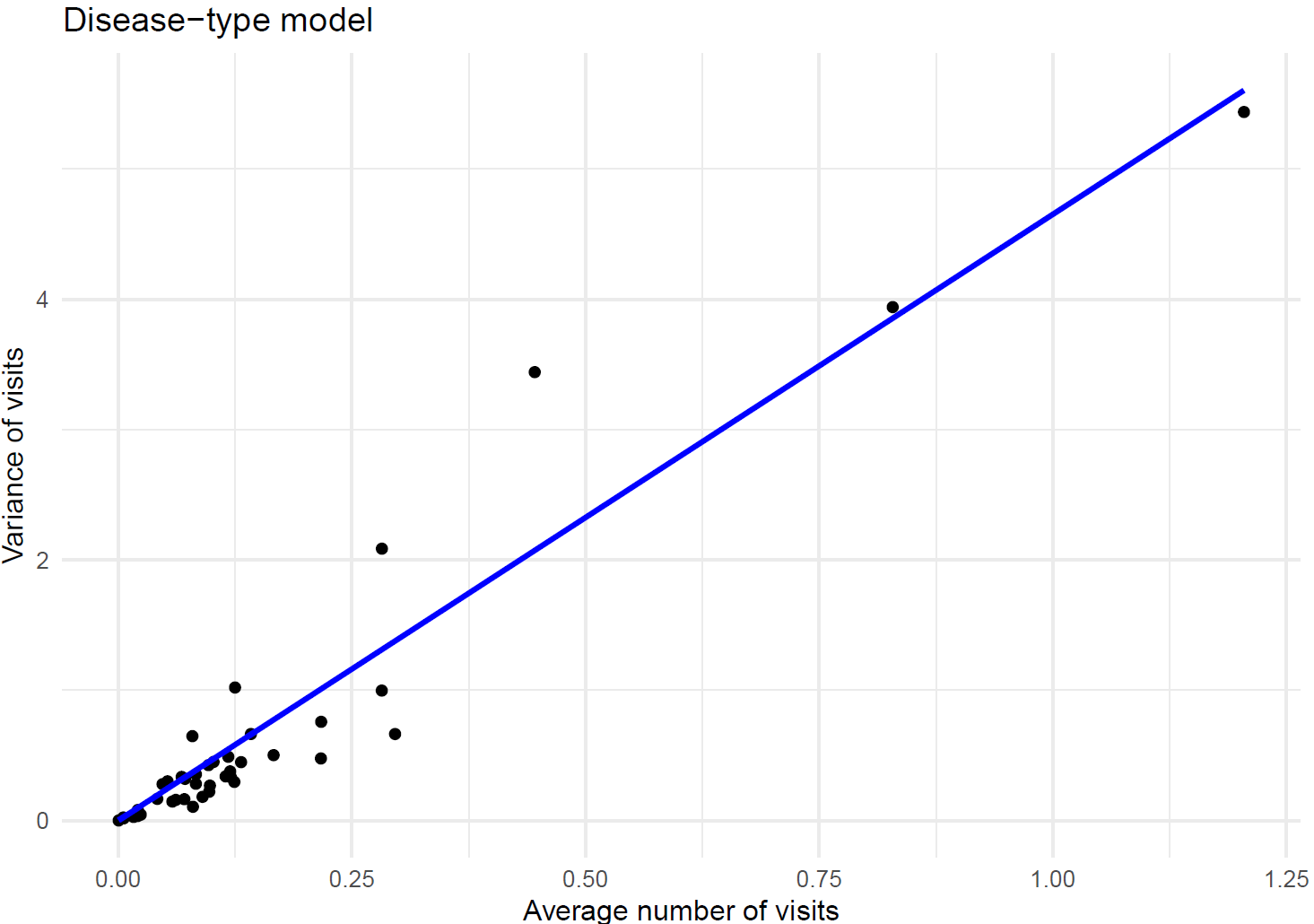


**Disease types, age groups, by sex (Figure 3b):** Same procedure as described above, but grouping by disease types, age groups, and sex. the result $\beta_{1}=3.8$ and $\beta_{2}=0.99$ ($R^{2}=0.926)$.


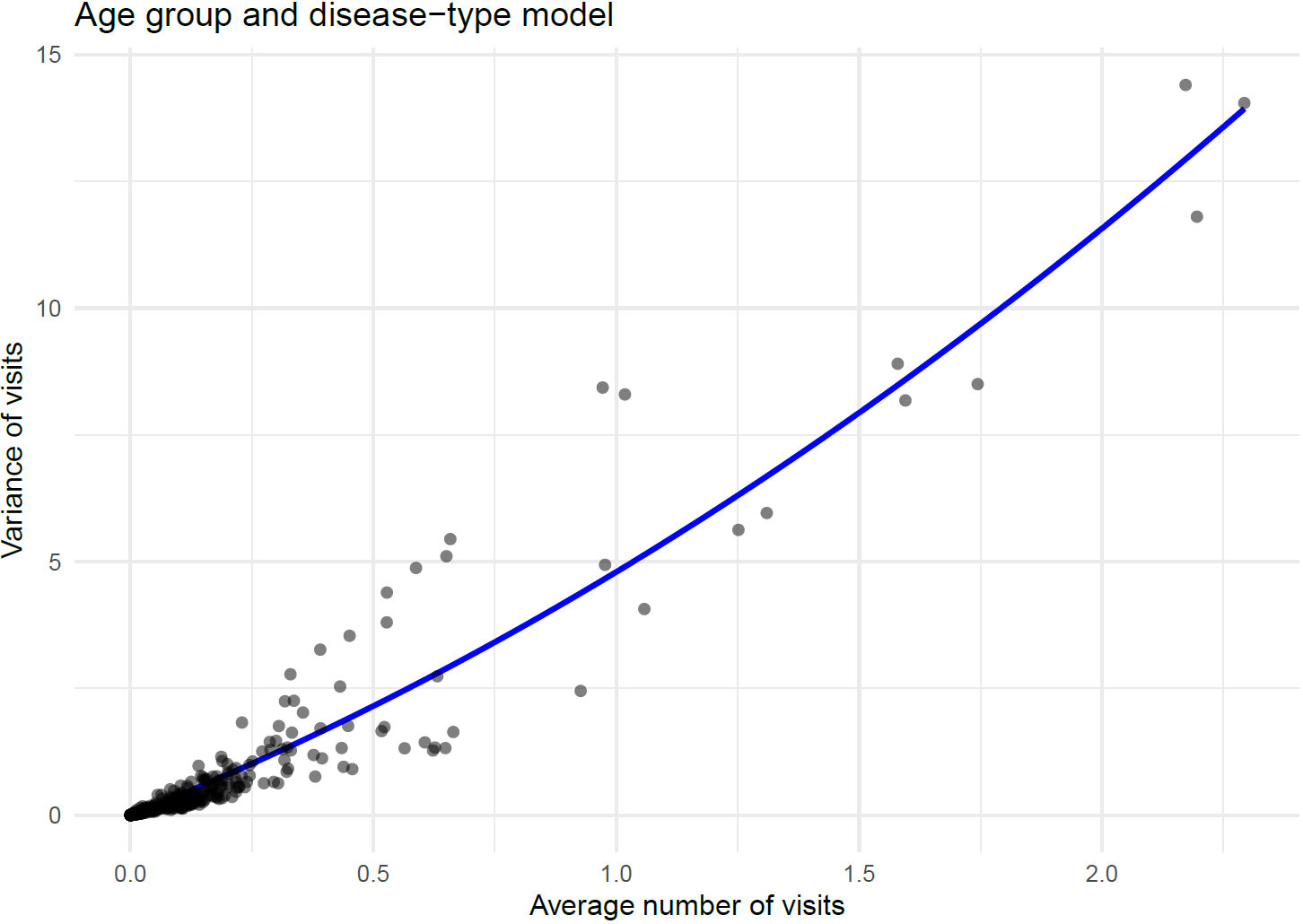


**Supplementary Table 1.** **Average number of GP consultations in primary care per inhabitant for different age groups in 2012 and 2023, by sex.**

| **Sex** | **Age group** | **Average 2012**  **(95% CI)** | **Average 2013**  **(95% CI)** | **Difference**  **2023-2012** | **p-value**  **difference** |
| --- | --- | --- | --- | --- | --- |
| Women | All ages | 3.09(3.08-3.1) | 3.58(3.57-3.59) | 0.49 | 0.000 |
| Men | All ages | 2.2(2.2-2.2) | 2.43(2.43-2.43) | 0.23 | 0.000 |
| Women | 0-5 years | 1.77(1.76-1.78) | 1.78(1.77-1.79) | 0.01 | 0.326 |
| Men | 0-5 years | 1.89(1.88-1.9) | 1.97(1.95-1.99) | 0.08 | 0.000 |
| Women | 6-15 years | 1.17(1.16-1.18) | 1.48(1.47-1.49) | 0.31 | 0.000 |
| Men | 6-15 years | 1.08(1.07-1.09) | 1.36(1.35-1.37) | 0.28 | 0.000 |
| Women | 16-19 years | 2.05(2.03-2.07) | 2.99(2.97-3.01) | 0.94 | 0.000 |
| Men | 16-19 years | 1.19(1.18-1.2) | 1.87(1.85-1.89) | 0.68 | 0.000 |
| Women | 20-29 years | 2.97(2.96-2.98) | 3.49(3.47-3.51) | 0.52 | 0.000 |
| Men | 20-29 years | 1.4(1.39-1.41) | 1.77(1.76-1.78) | 0.37 | 0.000 |
| Women | 30-49 years | 3.35(3.34-3.36) | 4.18(4.17-4.19) | 0.83 | 0.000 |
| Men | 30-49 years | 1.89(1.88-1.9) | 2.19(2.18-2.2) | 0.3 | 0.000 |
| Women | 50-66 years | 3.6(3.59-3.61) | 4.1(4.09-4.11) | 0.5 | 0.000 |
| Men | 50-66 years | 2.82(2.81-2.83) | 2.91(2.9-2.92) | 0.09 | 0.000 |
| Women | 67-79 years | 4.48(4.46-4.5) | 4.13(4.11-4.15) | -0.35 | 0.000 |
| Men | 67-79 years | 4.28(4.26-4.3) | 3.63(3.61-3.65) | -0.65 | 0.000 |
| Women | 80-89 years | 5(4.96-5.04) | 4.82(4.78-4.86) | -0.18 | 0.000 |
| Men | 80-89 years | 5.43(5.38-5.48) | 4.71(4.67-4.75) | -0.72 | 0.000 |
| Women | ≥90 years | 2.89(2.84-2.94) | 3.21(3.16-3.26) | 0.32 | 0.000 |
| Men | ≥90 years | 3.94(3.84-4.04) | 4(3.91-4.09) | 0.06 | 0.379 |

**Notes.** The table shows the average number of GP consultations in primary care per inhabitant for different age groups, by sex. Corresponds to Figure 1 in the manuscript.

**Supplementary Figure 1.** **Average number of yearly GP consultations in primary care per inhabitant for different age groups, by year and sex.**

**
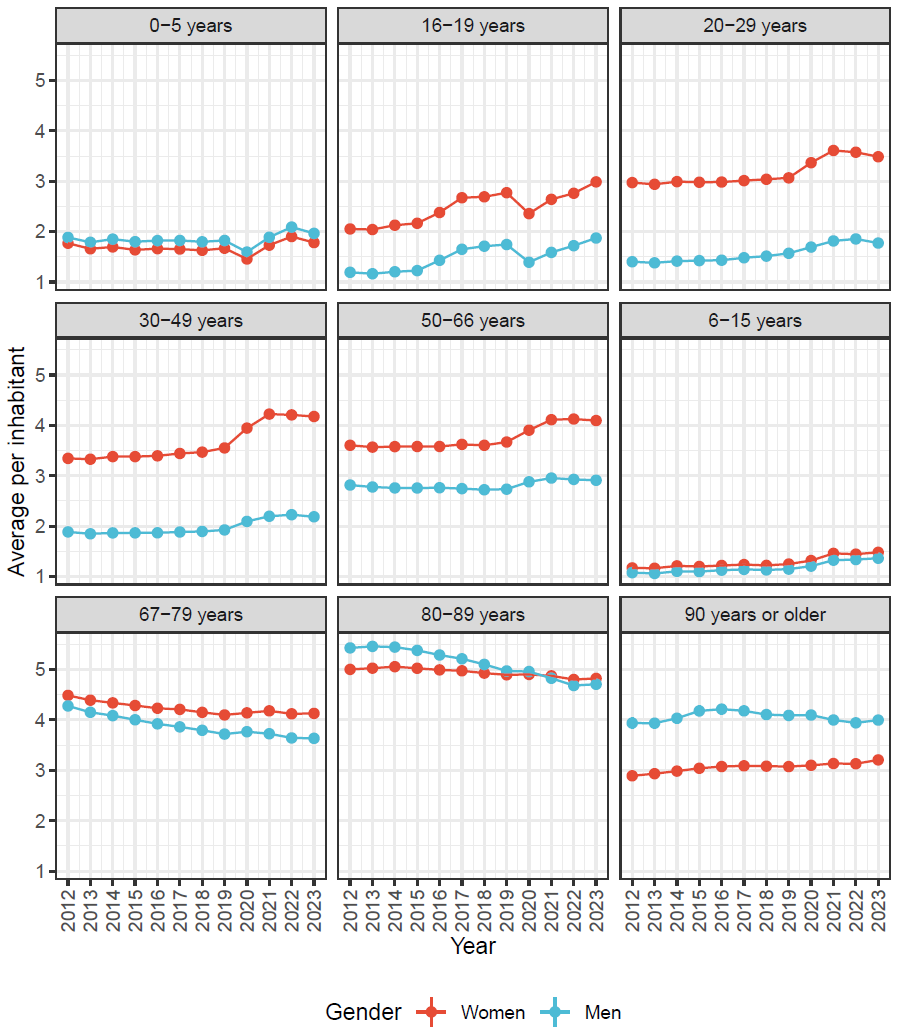
**

**Note.** Figure includes 95% Cis, but these are not visible, due to large sample sizes (covered by the points).

**Supplementary Table 2.** **Average number of GP consultations in primary care per inhabitant for different disease types in 2012 and 2023, by sex.**

| **Sex** | **Disease type** | **Average 2012**  **(95% CI)** | **Average 2023**  **(95% CI)** | **Difference**  **2023-2012** | **p-value** |
| --- | --- | --- | --- | --- | --- |
| Women | Administrative contact | 0.042(0.0415-0.0425) | 0.082(0.0813-0.0827) | 0.04 | 0 |
| Men | Administrative contact | 0.049(0.0484-0.0496) | 0.075(0.0743-0.0757) | 0.026 | 0 |
| Women | Atopy, asthma, allergy or eczema | 0.092(0.0912-0.0928) | 0.102(0.1012-0.1028) | 0.01 | 0 |
| Men | Atopy, asthma, allergy or eczema | 0.074(0.0733-0.0747) | 0.084(0.0833-0.0847) | 0.01 | 0 |
| Women | Diabetes | 0.059(0.0583-0.0597) | 0.07(0.0693-0.0707) | 0.011 | 0 |
| Men | Diabetes | 0.073(0.0723-0.0737) | 0.094(0.0932-0.0948) | 0.021 | 0 |
| Women | Preventive contact | 0.069(0.0683-0.0697) | 0.13(0.1291-0.1309) | 0.061 | 0 |
| Men | Preventive contact | 0.02(0.0196-0.0204) | 0.068(0.0673-0.0687) | 0.048 | 0 |
| Women | Health-related anxiety | 0.018(0.0176-0.0184) | 0.019(0.0186-0.0194) | 0.001 | 0.0001 |
| Men | Health-related anxiety | 0.014(0.0137-0.0143) | 0.014(0.0137-0.0143) | 0 | 1 |
| Women | Functional digestive issues | 0.12(0.1191-0.1209) | 0.155(0.154-0.156) | 0.035 | 0 |
| Men | Functional digestive issues | 0.072(0.0713-0.0727) | 0.093(0.0922-0.0938) | 0.021 | 0 |
| Women | General pain and muscle complaints | 0.055(0.0544-0.0556) | 0.058(0.0574-0.0586) | 0.003 | 0 |
| Men | General pain and muscle complaints | 0.018(0.0176-0.0184) | 0.019(0.0186-0.0194) | 0.001 | 0.00009 |
| Women | Heart disease | 0.117(0.1161-0.1179) | 0.048(0.0474-0.0486) | -0.069 | 0 |
| Men | Heart disease | 0.176(0.1749-0.1771) | 0.079(0.0783-0.0797) | -0.097 | 0 |
| Women | Skin infections | 0.052(0.0514-0.0526) | 0.055(0.0544-0.0556) | 0.003 | 0 |
| Men | Skin infections | 0.048(0.0474-0.0486) | 0.052(0.0514-0.0526) | 0.004 | 0 |
| Women | High blood pressure | 0.135(0.134-0.136) | 0.107(0.1062-0.1078) | -0.028 | 0 |
| Men | High blood pressure | 0.116(0.1151-0.1169) | 0.098(0.0972-0.0988) | -0.018 | 0 |
| Women | Cancer | 0.032(0.0315-0.0325) | 0.043(0.0425-0.0435) | 0.011 | 0 |
| Men | Cancer | 0.038(0.0375-0.0385) | 0.045(0.0445-0.0455) | 0.007 | 0 |
| Women | Joint and arthritic diseases | 0.076(0.0753-0.0767) | 0.063(0.0624-0.0636) | -0.013 | 0 |
| Men | Joint and arthritic diseases | 0.035(0.0345-0.0355) | 0.033(0.0325-0.0335) | -0.002 | 0 |
| Women | Local pain and inflammation | 0.242(0.2407-0.2433) | 0.26(0.2587-0.2613) | 0.018 | 0 |
| Men | Local pain and inflammation | 0.184(0.1829-0.1851) | 0.21(0.2088-0.2112) | 0.026 | 0 |
| Women | Respiratory infections, incl. ear inf. | 0.311(0.3095-0.3125) | 0.297(0.2956-0.2984) | -0.014 | 0 |
| Men | Respiratory infections, incl. ear inf. | 0.233(0.2317-0.2343) | 0.218(0.2168-0.2192) | -0.015 | 0 |
| Women | Congenital diseases or defects | 0.005(0.0048-0.0052) | 0.005(0.0048-0.0052) | 0 | 1 |
| Men | Congenital diseases or defects | 0.005(0.0048-0.0052) | 0.005(0.0048-0.0052) | 0 | 1 |
| Women | Mental illness or disorder | 0.309(0.3075-0.3105) | 0.46(0.4583-0.4617) | 0.151 | 0 |
| Men | Mental illness or disorder | 0.202(0.2008-0.2032) | 0.276(0.2747-0.2773) | 0.074 | 0 |
| Women | Back problems | 0.101(0.1001-0.1019) | 0.112(0.1111-0.1129) | 0.011 | 0 |
| Men | Back problems | 0.088(0.0872-0.0888) | 0.104(0.1032-0.1048) | 0.016 | 0 |
| Women | Pregnancy, childbirth, contraception | 0.216(0.2148-0.2172) | 0.185(0.1839-0.1861) | -0.031 | 0 |
| Men | Pregnancy, childbirth, contraception | - | - | - | - |
| Women | Accidents and injuries | 0.08(0.0792-0.0808) | 0.089(0.0882-0.0898) | 0.009 | 0 |
| Men | Accidents and injuries | 0.09(0.0892-0.0908) | 0.088(0.0872-0.0888) | -0.002 | 0.00037 |
| Women | Gynecological issues | 0.071(0.0703-0.0717) | 0.102(0.1012-0.1028) | 0.031 | 0 |
| Men | Gynecological issues | - | - | - | - |

**Supplementary Figure 2.** **Average number of GP consultations in primary care per inhabitant for different age groups and year, by sex.**


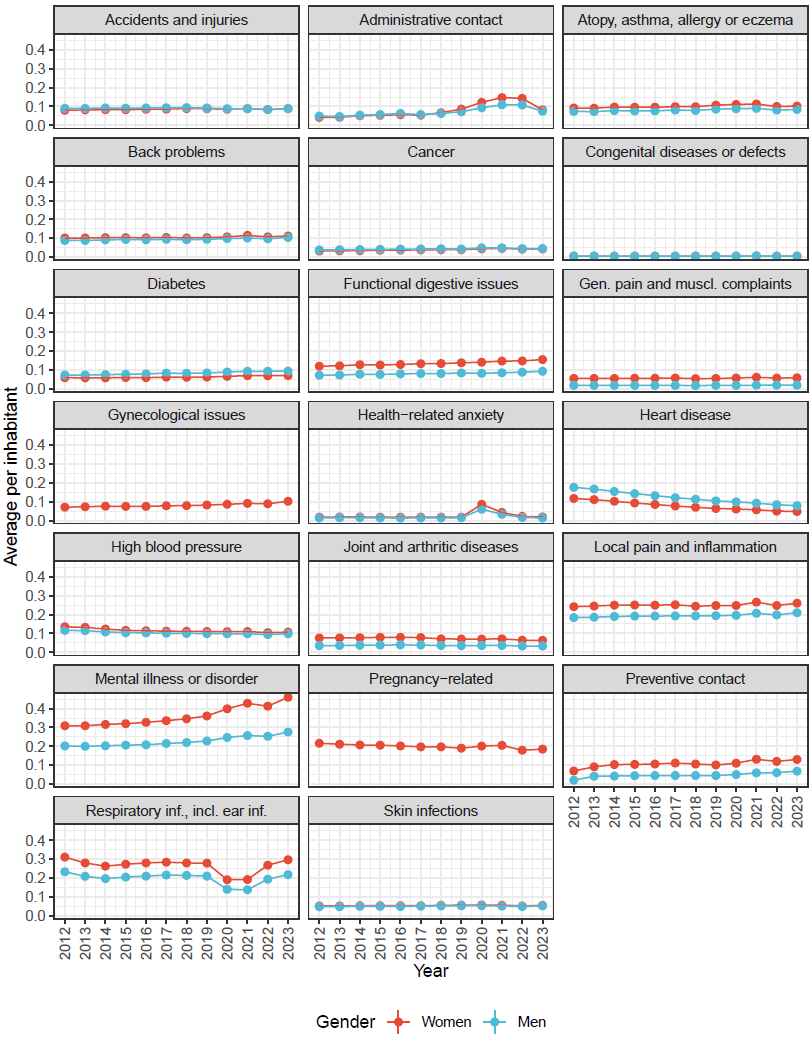

Supplement: Supplementary Material.docx [file IPRI_A_2666623_SM2264.docx]
