# Supplementary material for: General practitioner utilization by age, sex and disease in Norway from 2012–2023: a national health registry study
Source: Scand J Prim Health Care. 2026 May 11;44(1):2666623. doi: 10.1080/02813432.2026.2666623 (PMC13162542; doi:10.1080/02813432.2026.2666623)
Supplement: Online Supplementary.pdf [file IPRI_A_2666623_SM2263.pdf]

Online Supplementary Material

This material displays age-, sex-, and disease-type specific trends in general practitioner consultations. Please see Supplementary Material A for further details.

Table of Contents

Accidents and injuries **2**

Administrative contact **3**

Atopy, asthma, allergy or eczema **4**

Back problems **5**

Cancer **6**

Congenital diseases or defects **7**

Diabetes **8**

Functional digestive issues **9**

Gynecological issues **10**

Heart disease **11**

High blood pressure **12**

Health-related anxiety **13**

Joint and arthritic diseases **14**

Local pain and inflammation **15**

Mental illness or disorder **16**

Other diagnoses **17**

Preventive contact **18**

Pregnancy, childbirth, contraception **19**

Respiratory infections, including ear infections **20**

Skin infections **21**

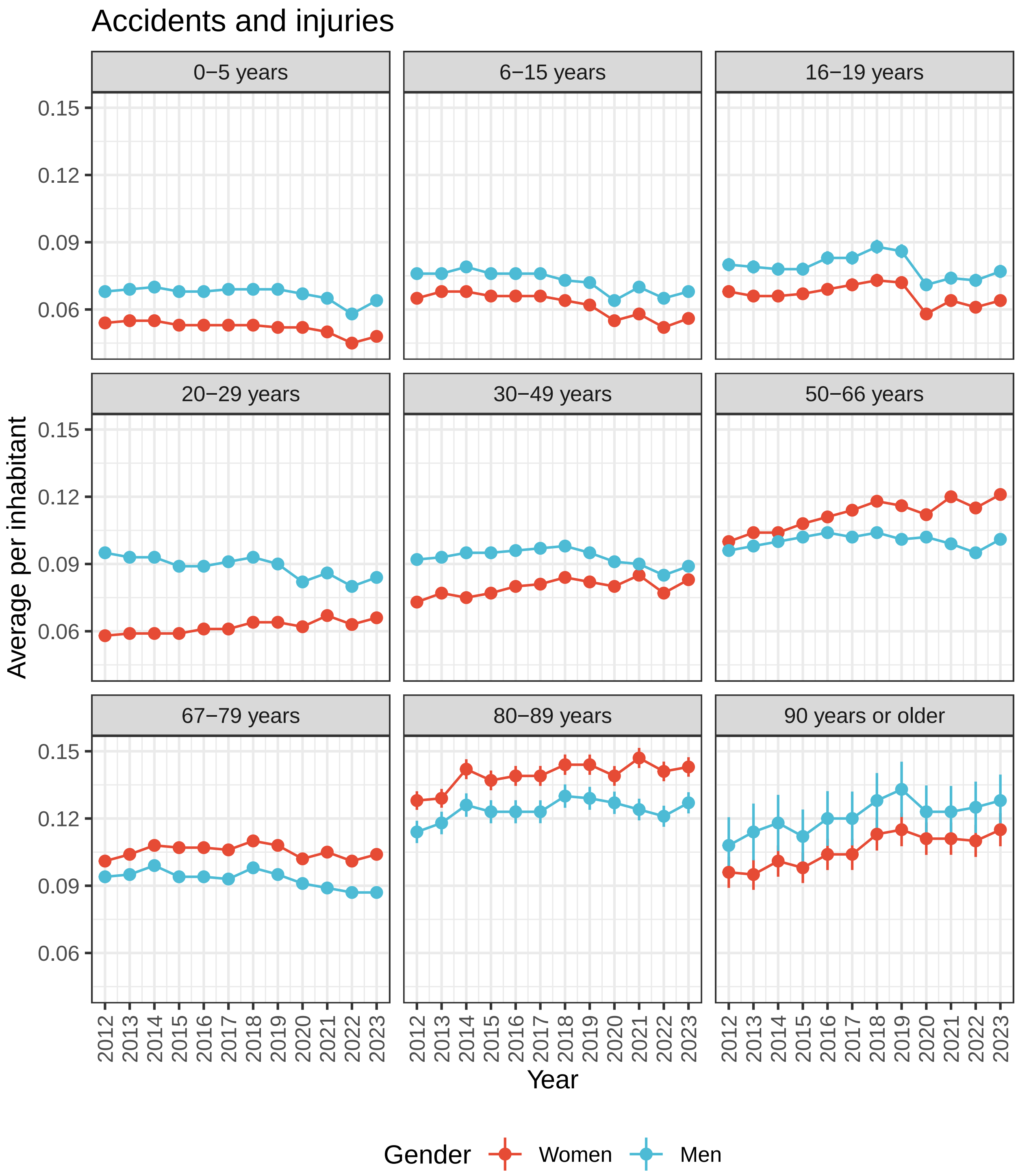
Accidents and injuries
0−5 years
6−15 years
16−19 years
20−29 years
30−49 years
50−66 years
67−79 years
80−89 years
90 years or older
Average per inhabitant
0.15
0.12
0.09
0.06
2012
2013
2014
2015
2016
2017
2018
2019
2020
2021
2022
2023
Year
Gender
Women
Men

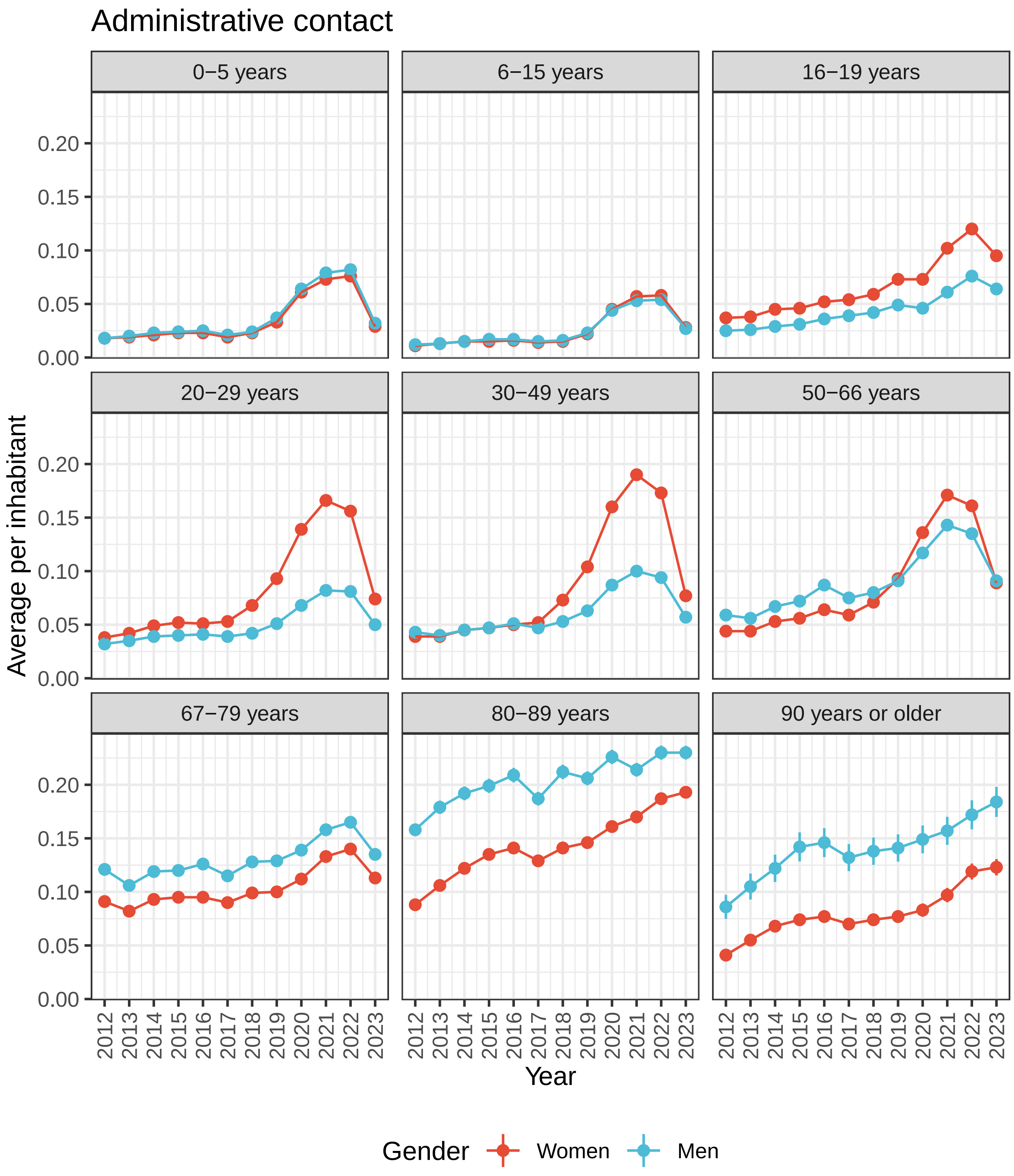
Administrative contact
0−5 years
6−15 years
16−19 years
20−29 years
30−49 years
50−66 years
67−79 years
80−89 years
90 years or older
Average per inhabitant
0.00
0.05
0.10
0.15
0.20
Year
2012
2013
2014
2015
2016
2017
2018
2019
2020
2021
2022
2023
Gender
Women
Men

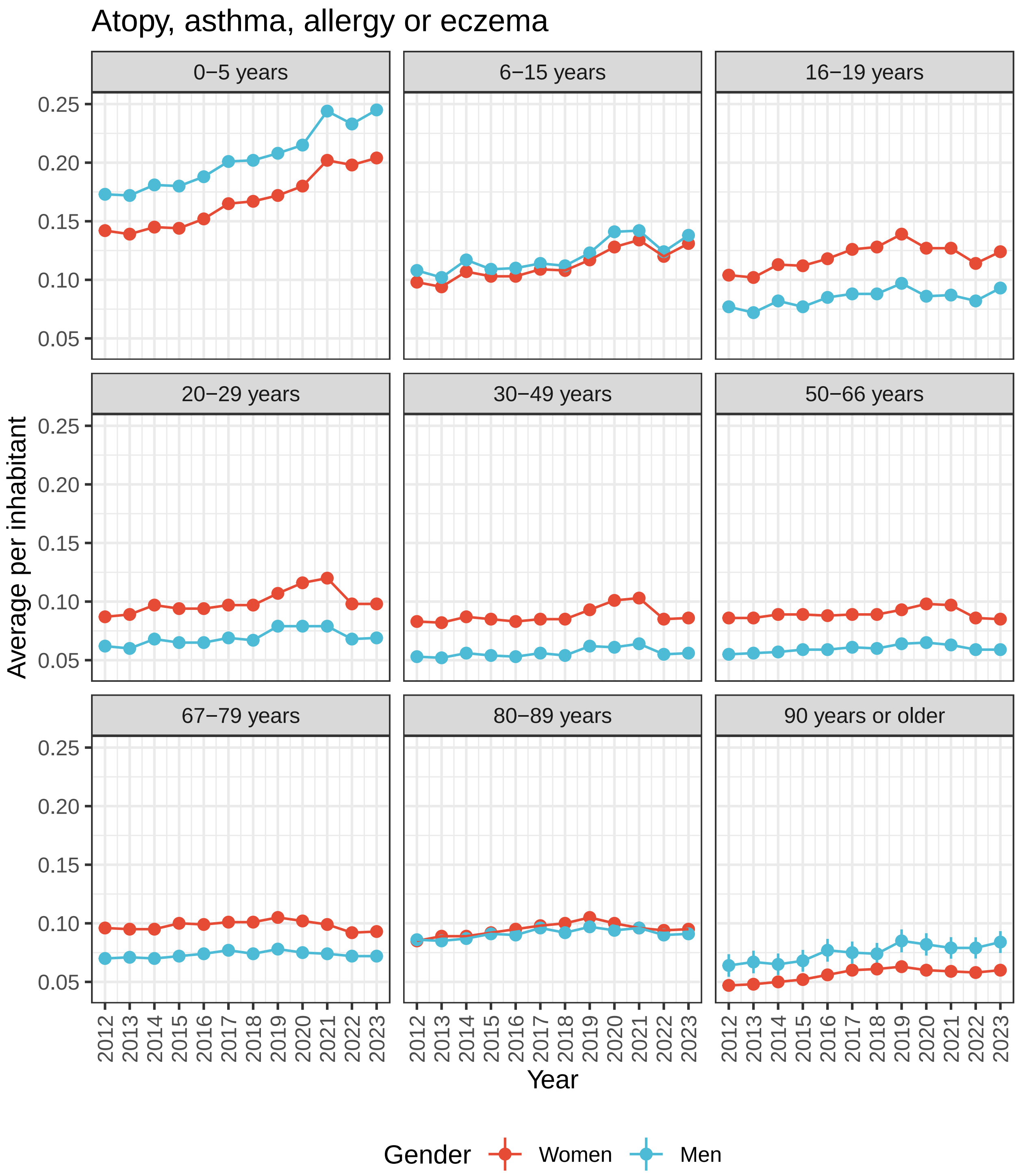

Atopy, asthma, allergy or eczema
0−5 years
6−15 years
16−19 years
20−29 years
30−49 years
50−66 years
67−79 years
80−89 years
90 years or older
Average per inhabitant
0.25
0.20
0.15
0.10
0.05
2012
2013
2014
2015
2016
2017
2018
2019
2020
2021
2022
2023
Year
Gender
Women
Men

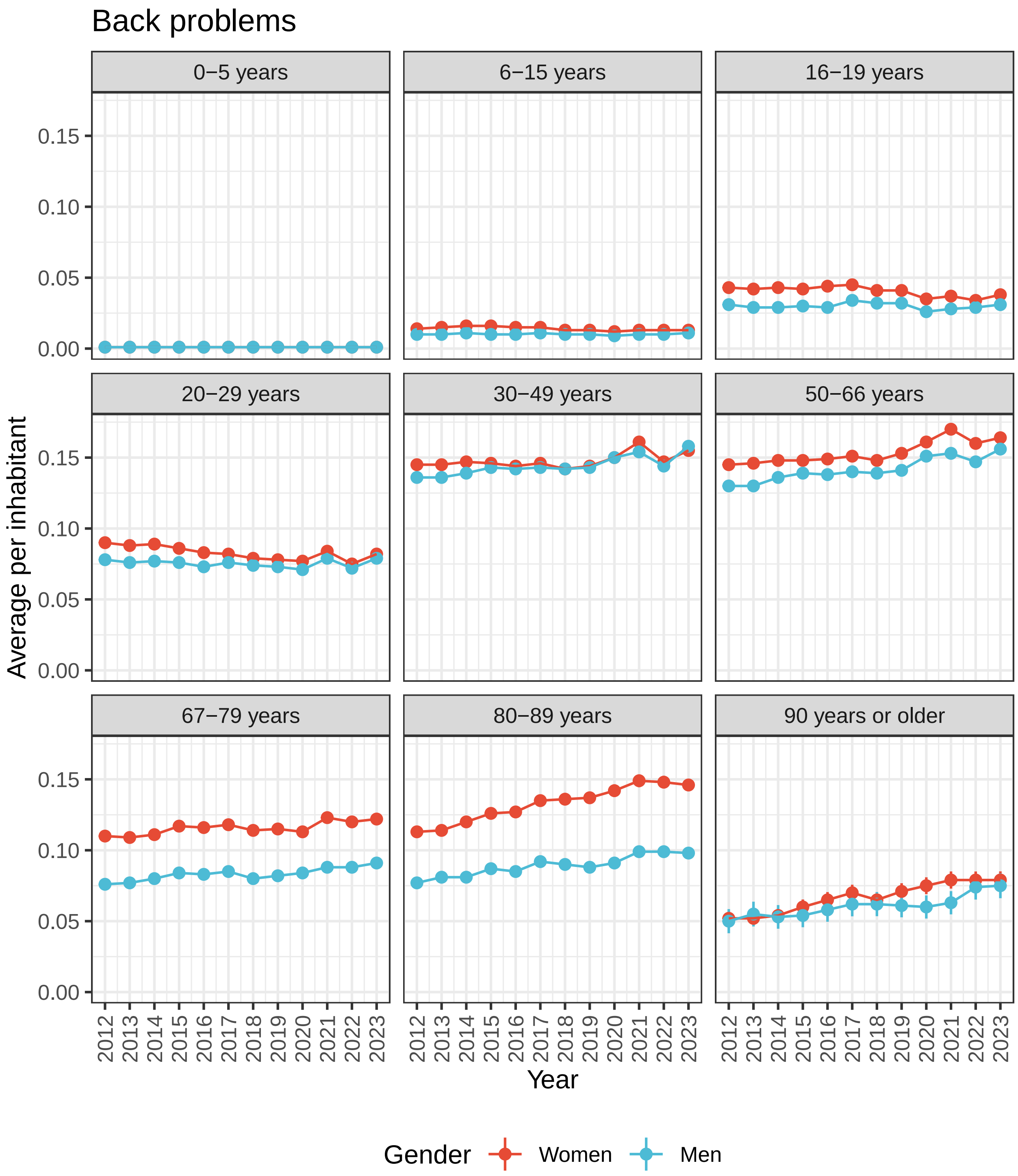

Back problems
0−5 years
6−15 years
16−19 years
20−29 years
30−49 years
50−66 years
67−79 years
80−89 years
90 years or older
Average per inhabitant
0.00
0.05
0.10
0.15
Year
2012
2013
2014
2015
2016
2017
2018
2019
2020
2021
2022
2023
Gender
Women
Men

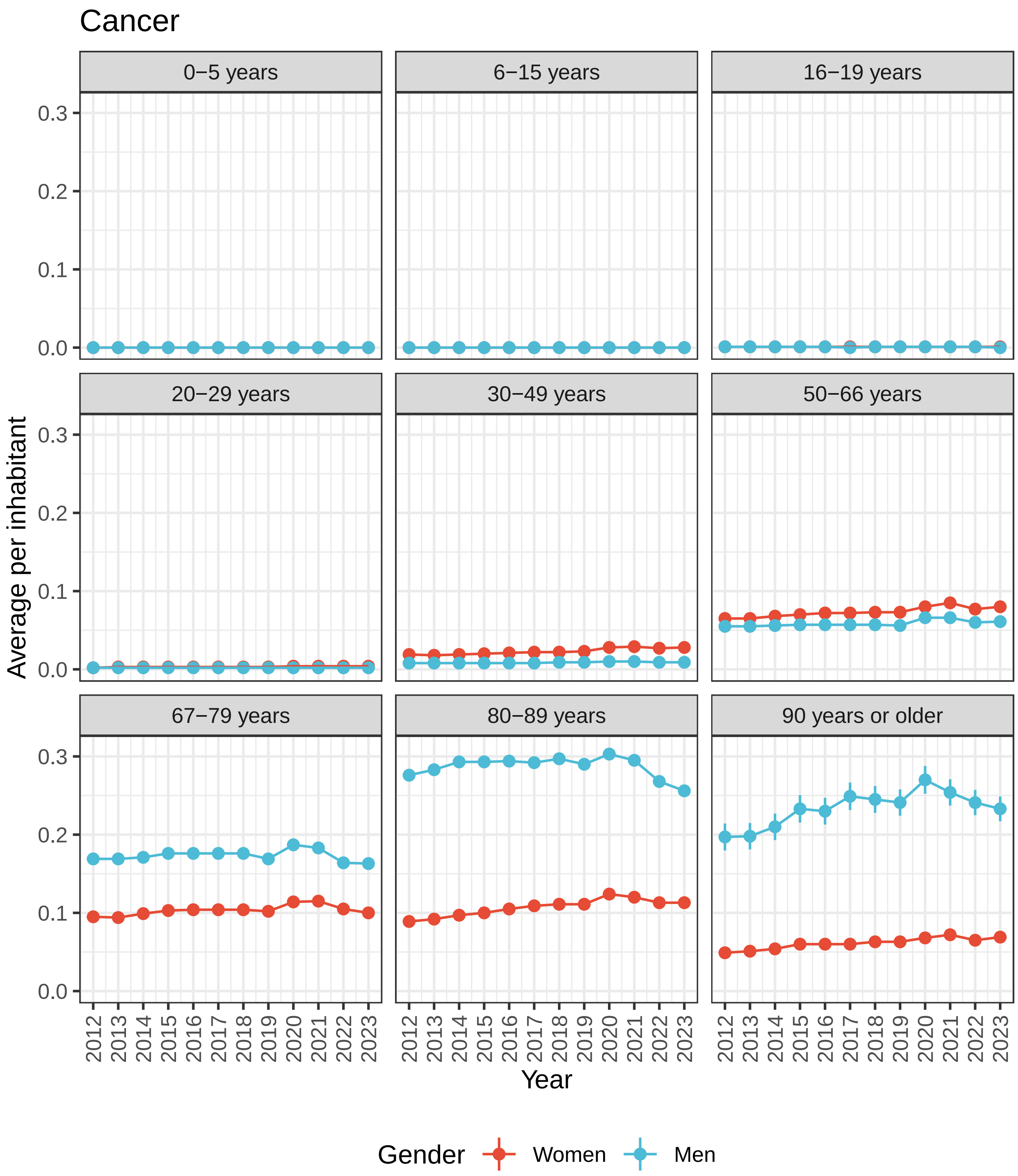

Cancer
0−5 years
6−15 years
16−19 years
20−29 years
30−49 years
50−66 years
67−79 years
80−89 years
90 years or older
Average per inhabitant
0.0
0.1
0.2
0.3
2012
2013
2014
2015
2016
2017
2018
2019
2020
2021
2022
2023
Year
Gender
Women
Men

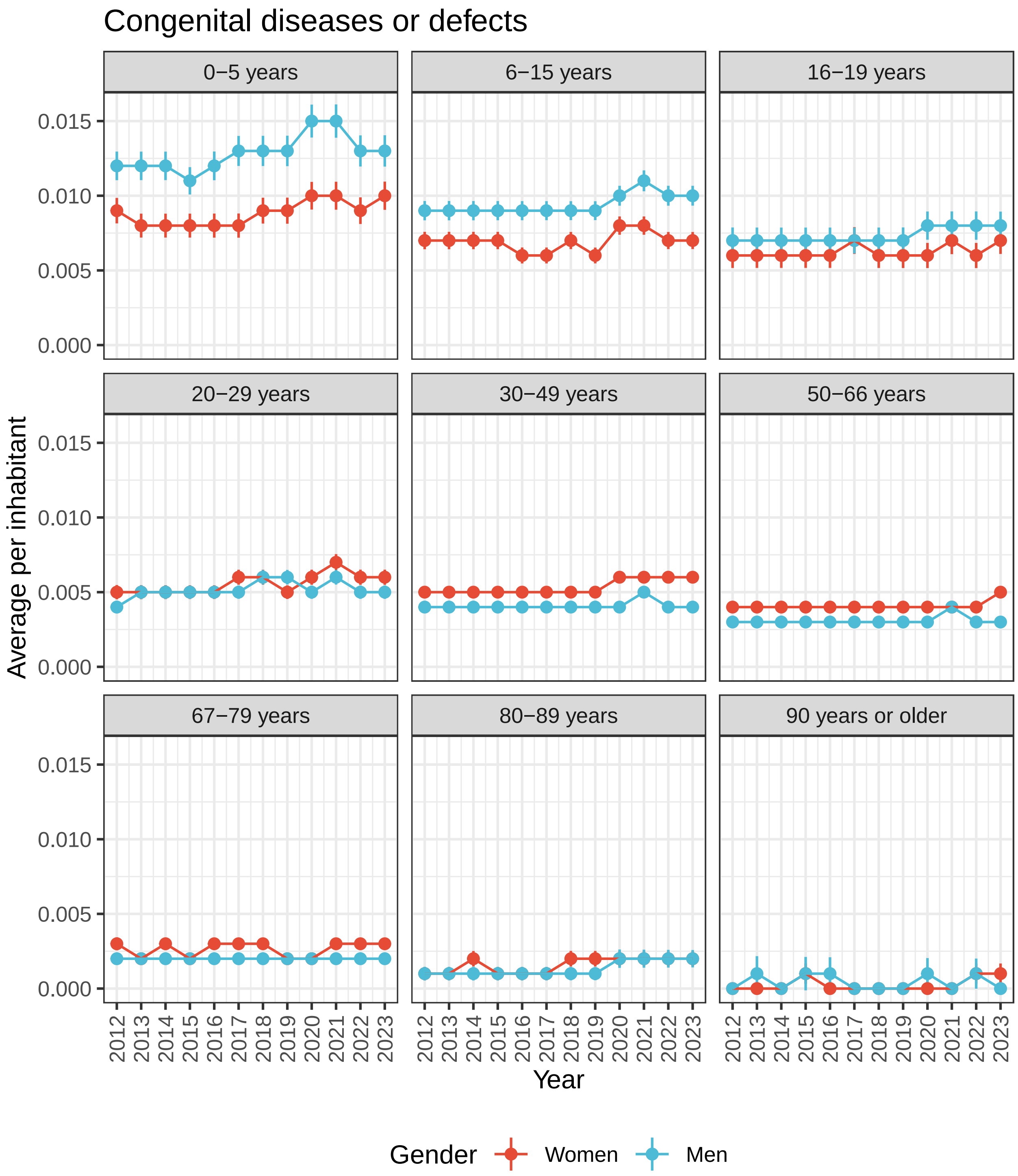

Congenital diseases or defects
0−5 years
6−15 years
16−19 years
20−29 years
30−49 years
50−66 years
67−79 years
80−89 years
90 years or older
Average per inhabitant
0.000
0.005
0.010
0.015
2012
2013
2014
2015
2016
2017
2018
2019
2020
2021
2022
2023
Year
Gender
Women
Men

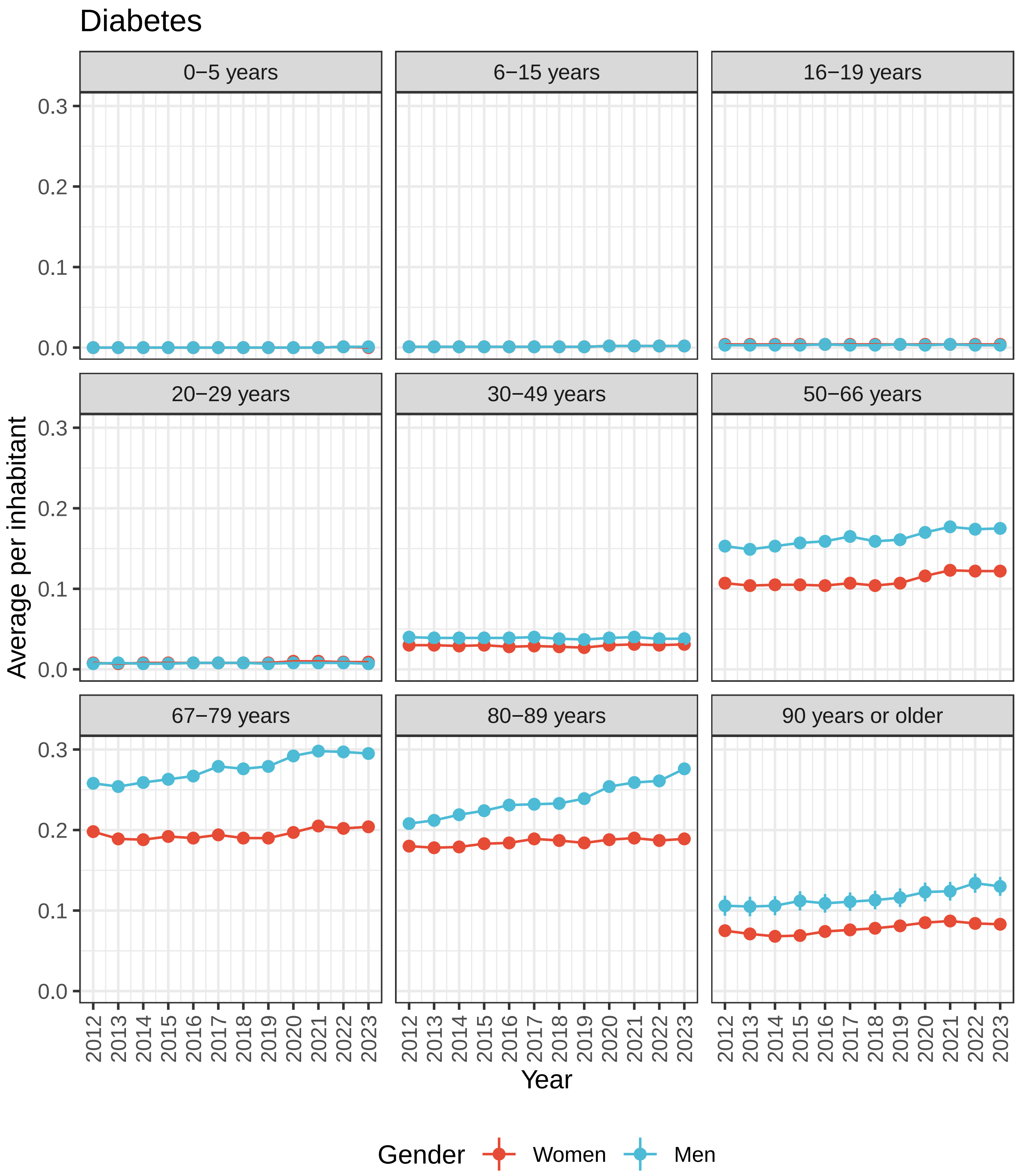
Diabetes
0−5 years
6−15 years
16−19 years
20−29 years
30−49 years
50−66 years
67−79 years
80−89 years
90 years or older
Average per inhabitant
0.0
0.1
0.2
0.3
2012
2013
2014
2015
2016
2017
2018
2019
2020
2021
2022
2023
Year
Gender
Women
Men

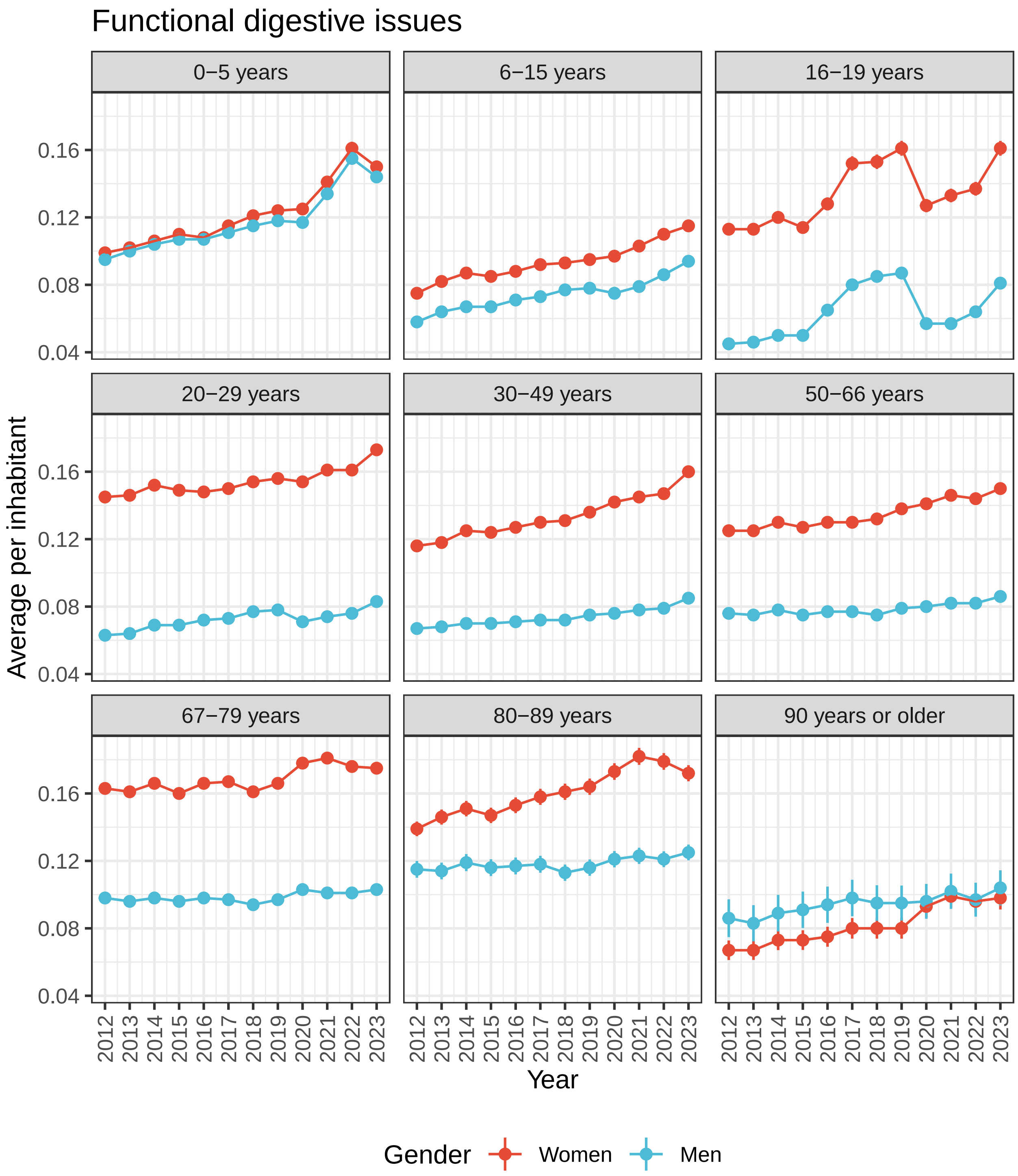
Functional digestive issues
0−5 years
6−15 years
16−19 years
20−29 years
30−49 years
50−66 years
67−79 years
80−89 years
90 years or older
Average per inhabitant
0.04
0.08
0.12
0.16
2012
2013
2014
2015
2016
2017
2018
2019
2020
2021
2022
2023
Year
Gender
Women
Men

Gynecological issues

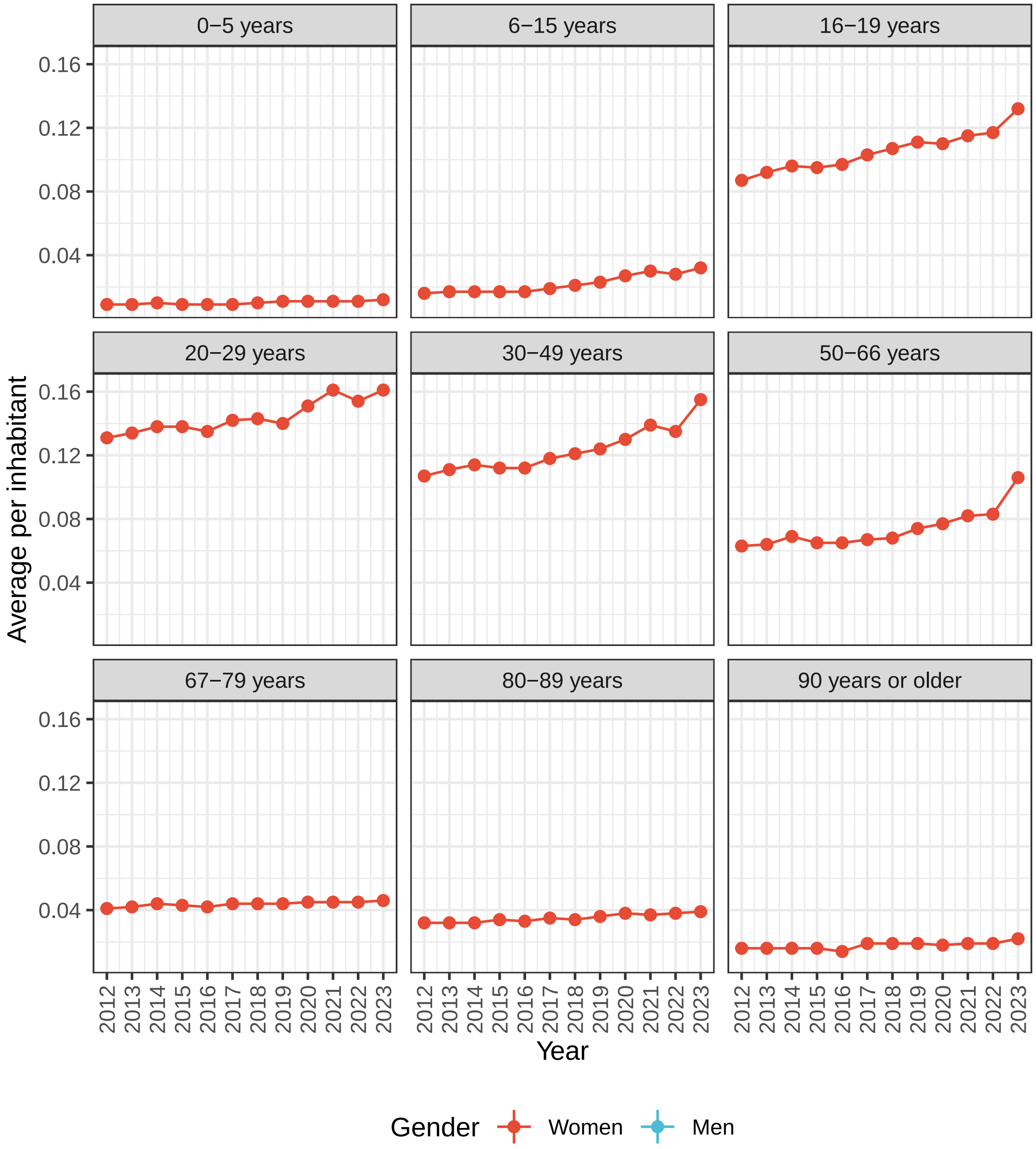

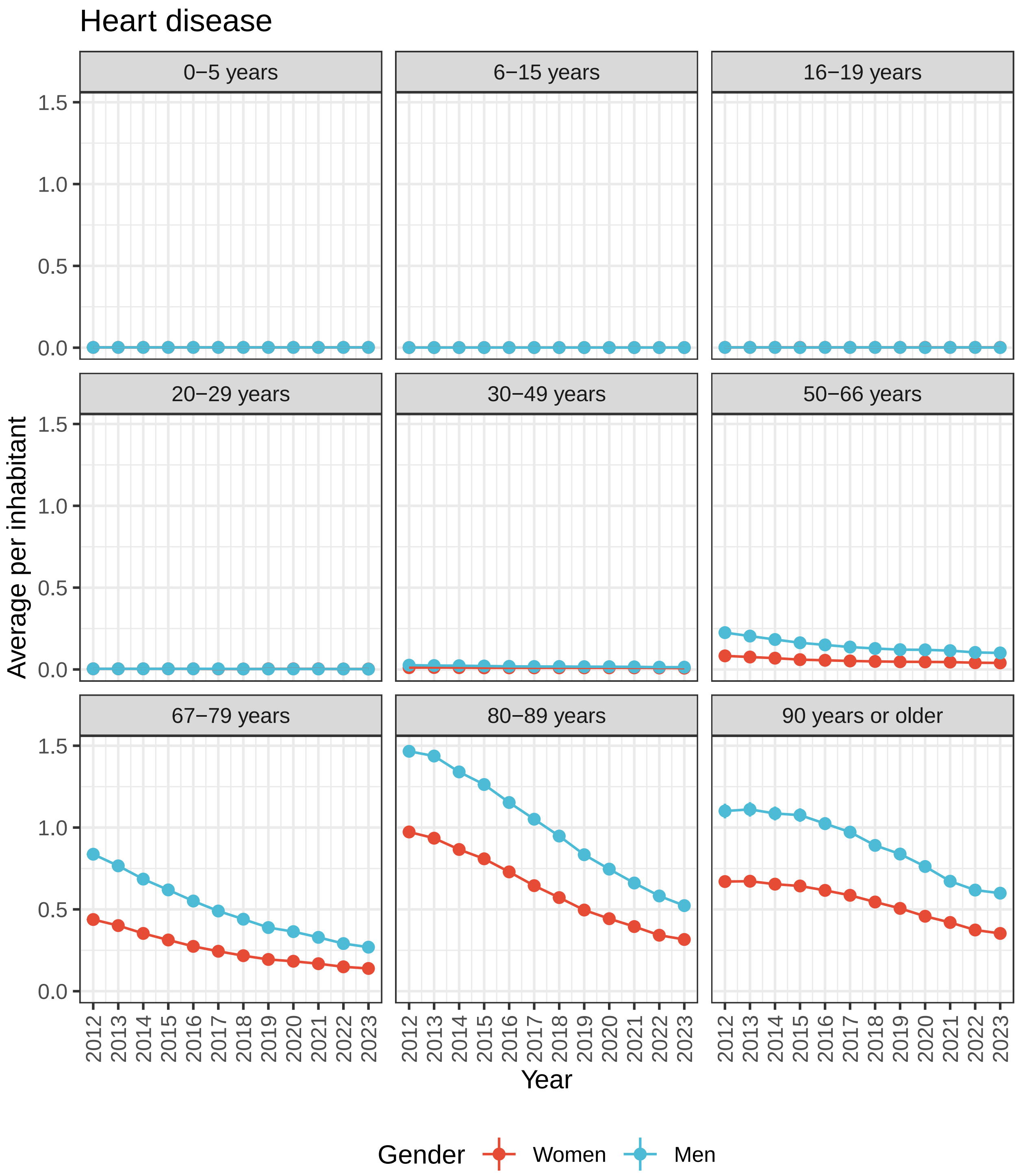
Heart disease
0−5 years
6−15 years
16−19 years
20−29 years
30−49 years
50−66 years
67−79 years
80−89 years
90 years or older
Average per inhabitant
0.0
0.5
1.0
1.5
2012
2013
2014
2015
2016
2017
2018
2019
2020
2021
2022
2023
Year
Gender
Women
Men

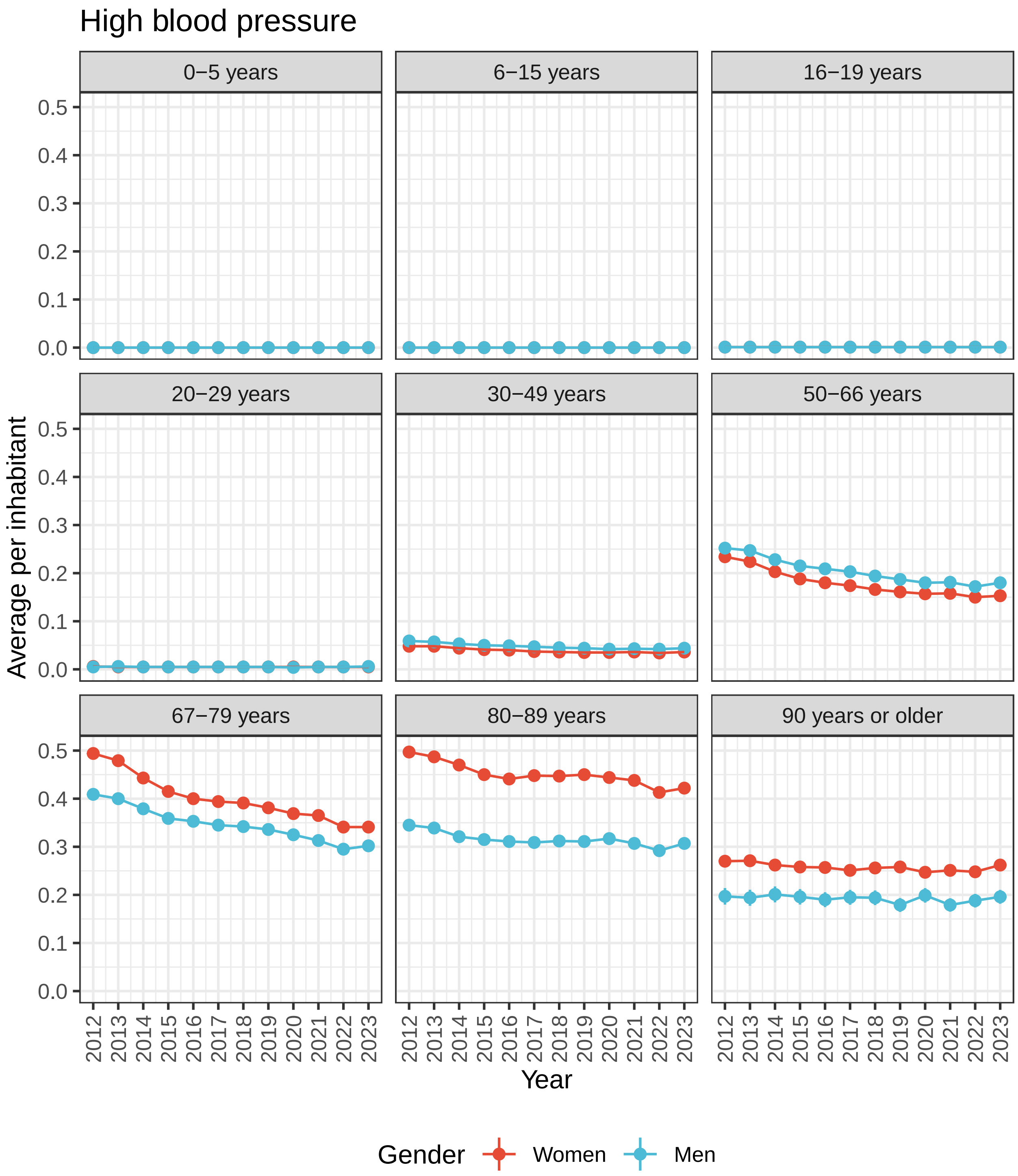
High blood pressure
0−5 years
6−15 years
16−19 years
20−29 years
30−49 years
50−66 years
67−79 years
80−89 years
90 years or older
Average per inhabitant
0.0
0.1
0.2
0.3
0.4
0.5
2012 2013 2014 2015 2016 2017 2018 2019 2020 2021 2022 2023
Year
Gender
Women
Men

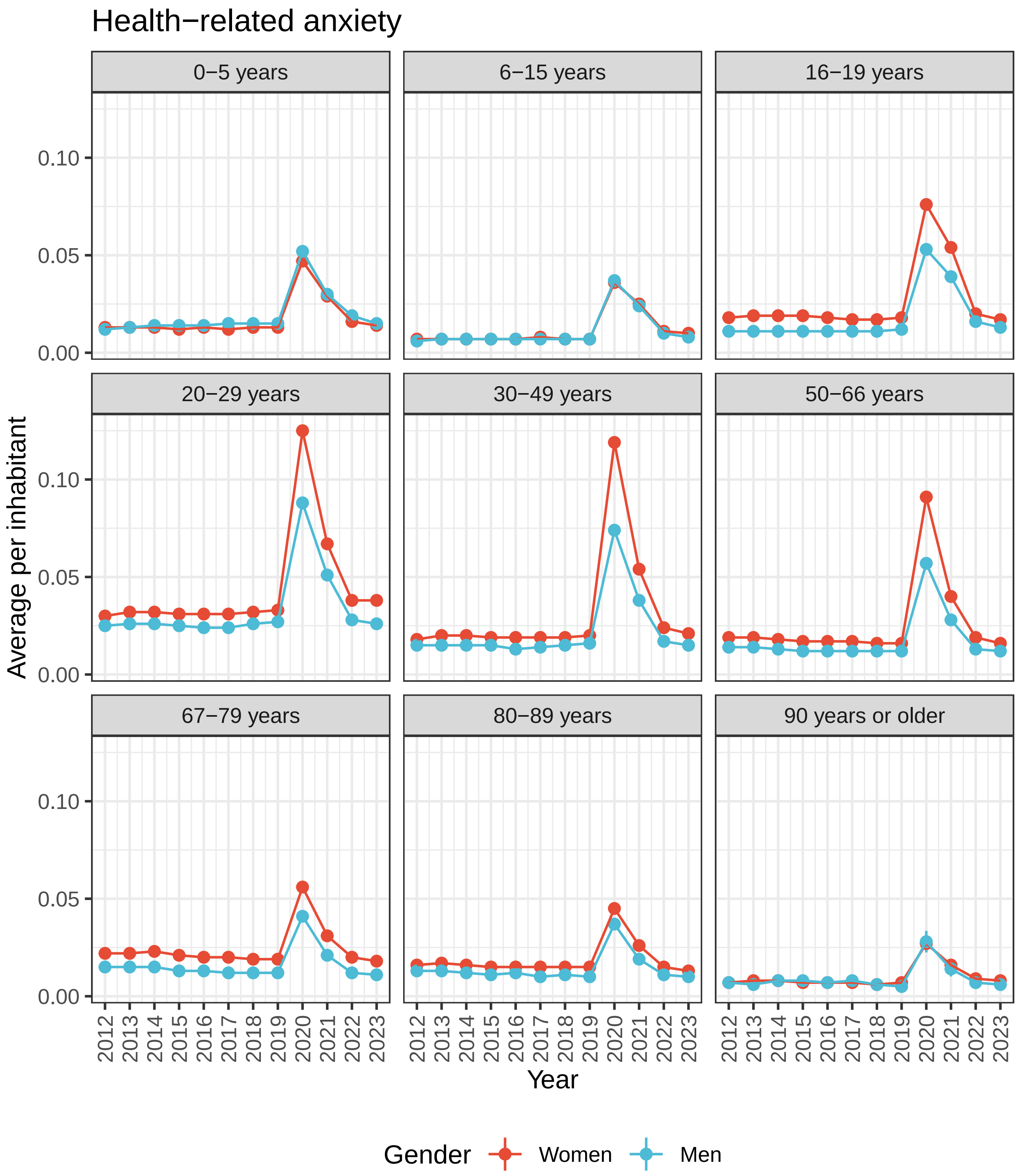

Health−related anxiety
0−5 years
6−15 years
16−19 years
20−29 years
30−49 years
50−66 years
67−79 years
80−89 years
90 years or older
Average per inhabitant
0.00
0.05
0.10
Year
2012
2013
2014
2015
2016
2017
2018
2019
2020
2021
2022
2023
Gender
Women
Men

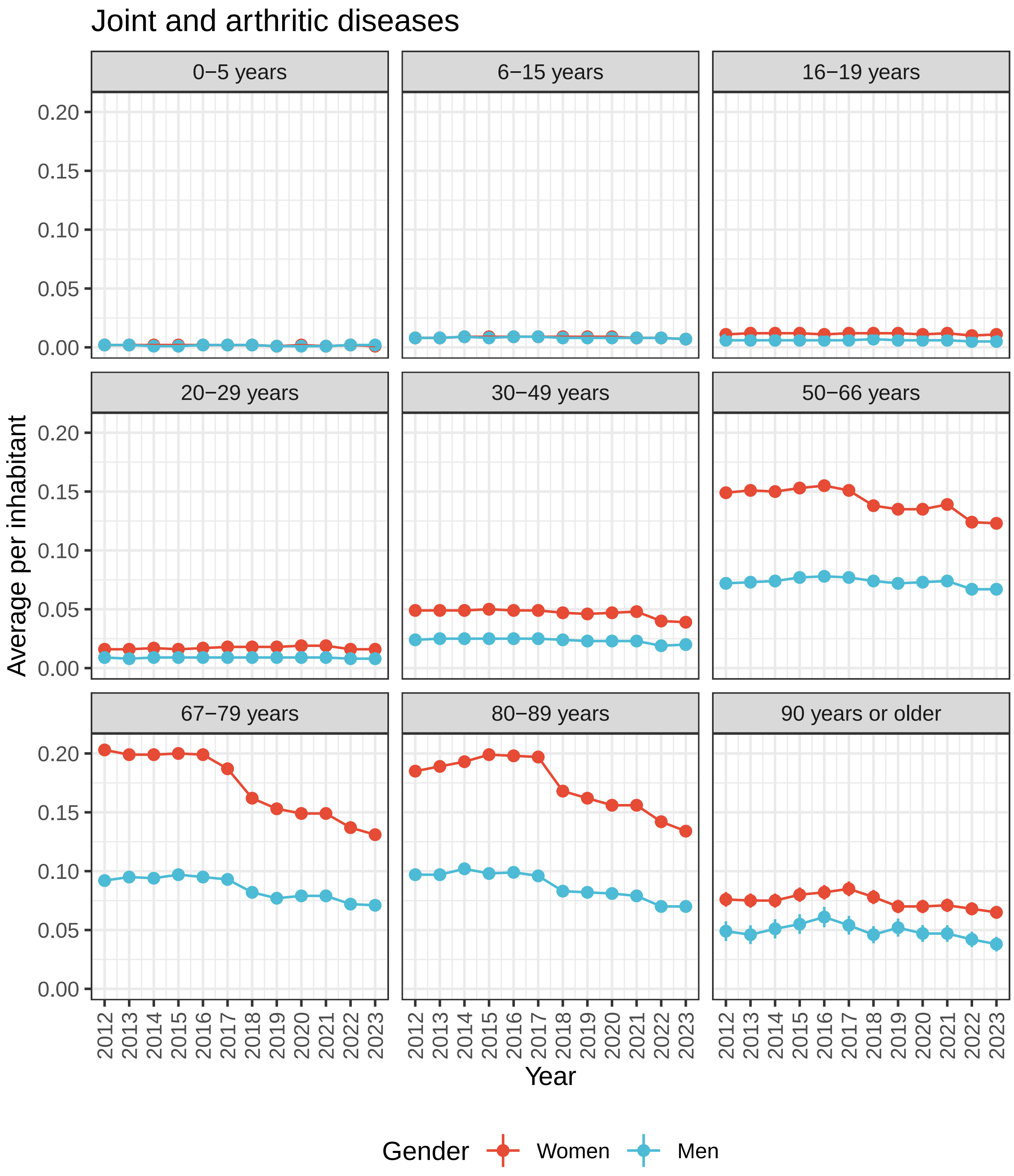

Joint and arthritic diseases
0−5 years
6−15 years
16−19 years
20−29 years
30−49 years
50−66 years
67−79 years
80−89 years
90 years or older
0.00
0.05
0.10
0.15
0.20
2012
2013
2014
2015
2016
2017
2018
2019
2020
2021
2022
2023
Average per inhabitant
Year
Gender
Women
Men

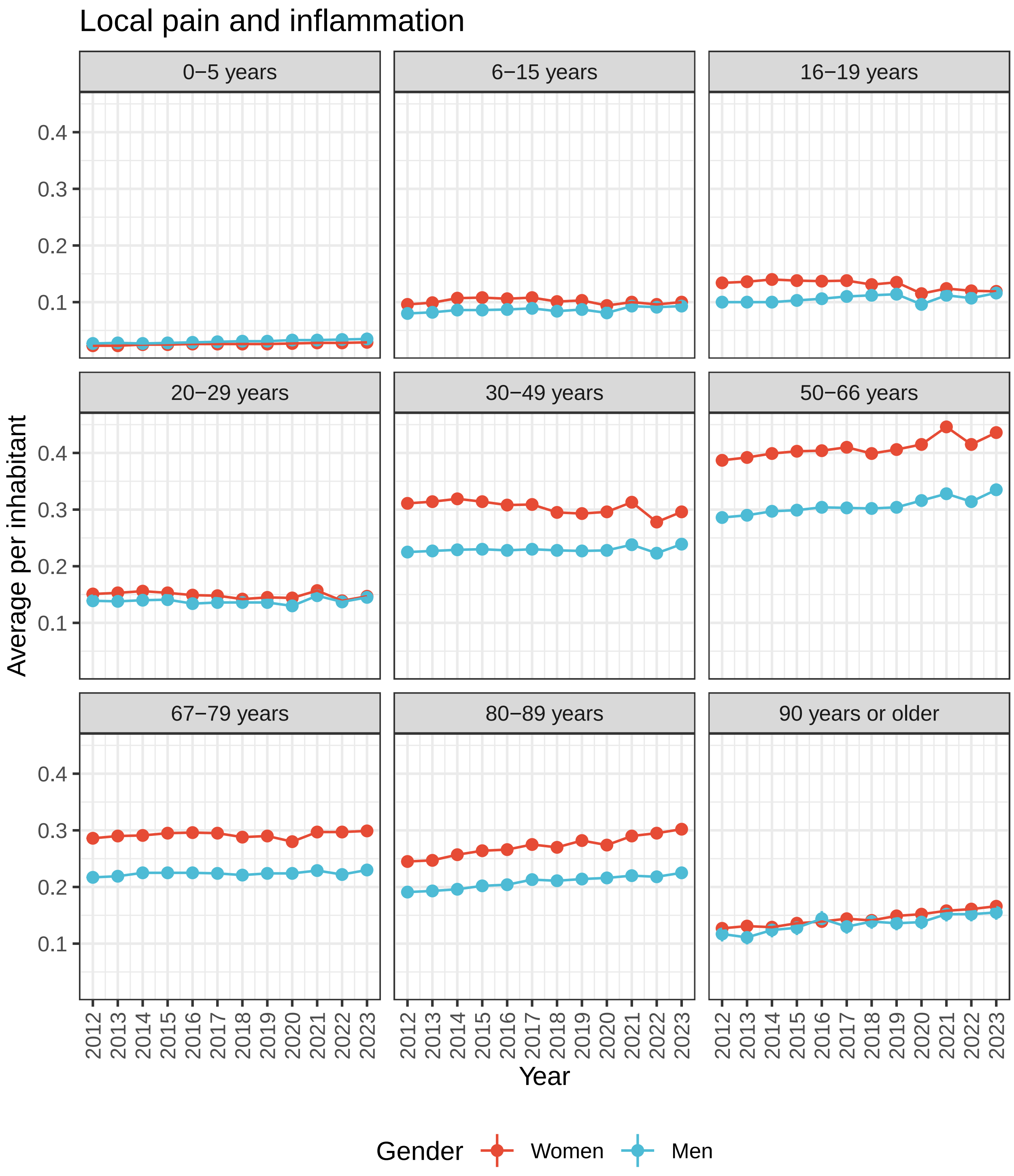

Local pain and inflammation
0−5 years
6−15 years
16−19 years
20−29 years
30−49 years
50−66 years
67−79 years
80−89 years
90 years or older
Average per inhabitant
0.1
0.2
0.3
0.4
2012
2013
2014
2015
2016
2017
2018
2019
2020
2021
2022
2023
Year
Gender
Women
Men

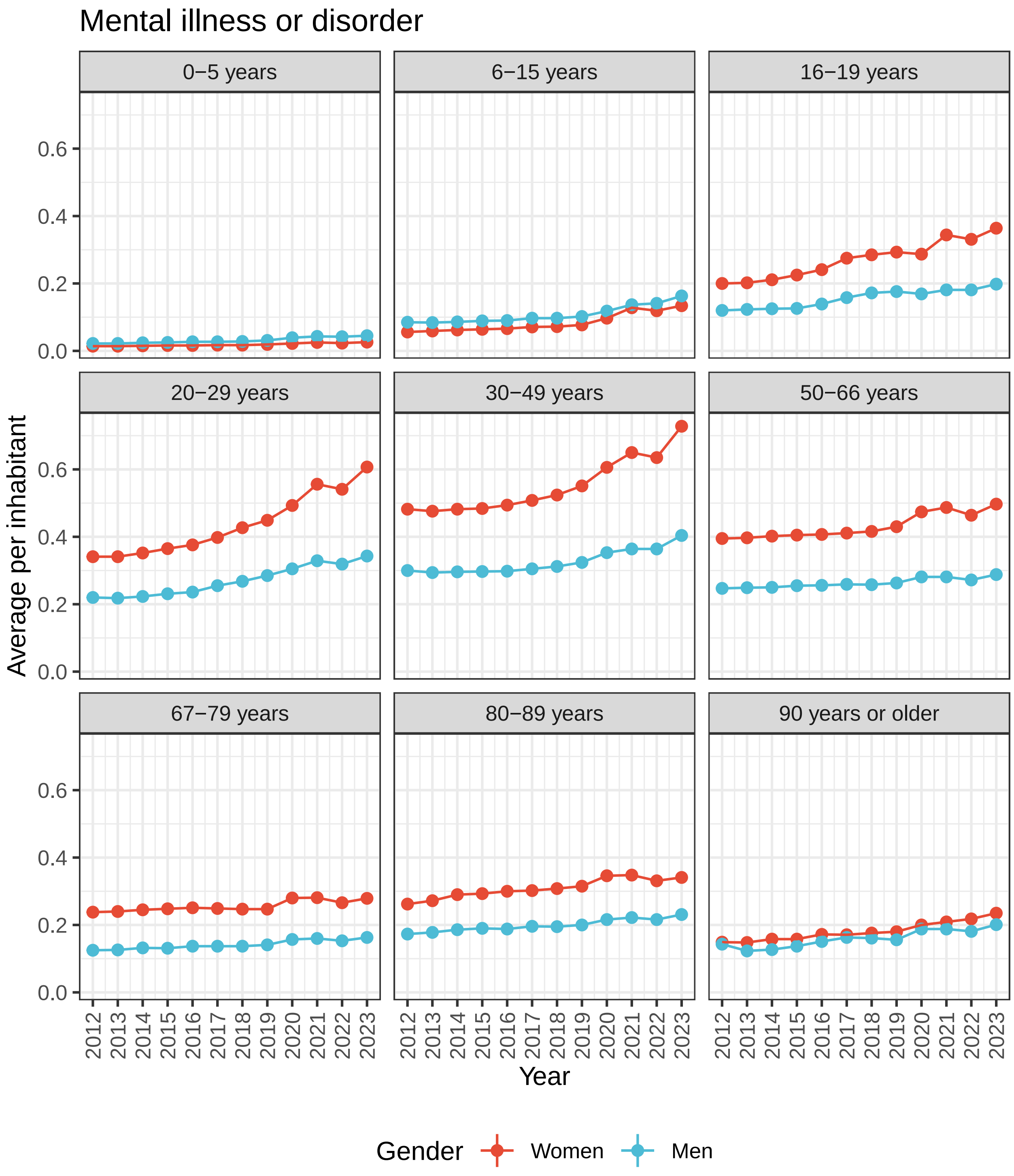

Mental illness or disorder
0−5 years
6−15 years
16−19 years
20−29 years
30−49 years
50−66 years
67−79 years
80−89 years
90 years or older
Average per inhabitant
0.0
0.2
0.4
0.6
2012
2013
2014
2015
2016
2017
2018
2019
2020
2021
2022
2023
Year
Gender
Women
Men

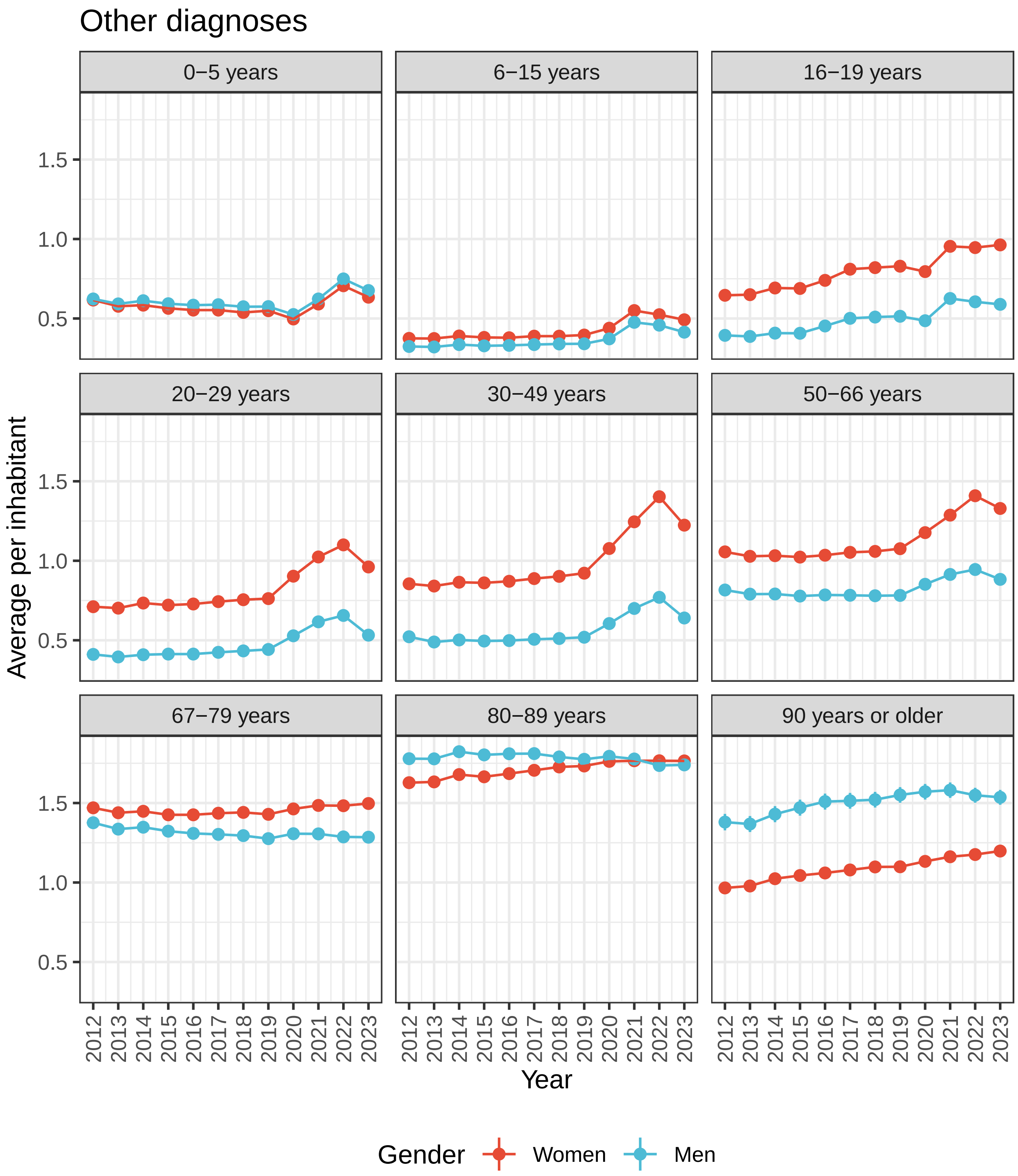

Other diagnoses
0−5 years
6−15 years
16−19 years
20−29 years
30−49 years
50−66 years
67−79 years
80−89 years
90 years or older
Average per inhabitant
0.5
1.0
1.5
Year
2012
2013
2014
2015
2016
2017
2018
2019
2020
2021
2022
2023
Gender
Women
Men

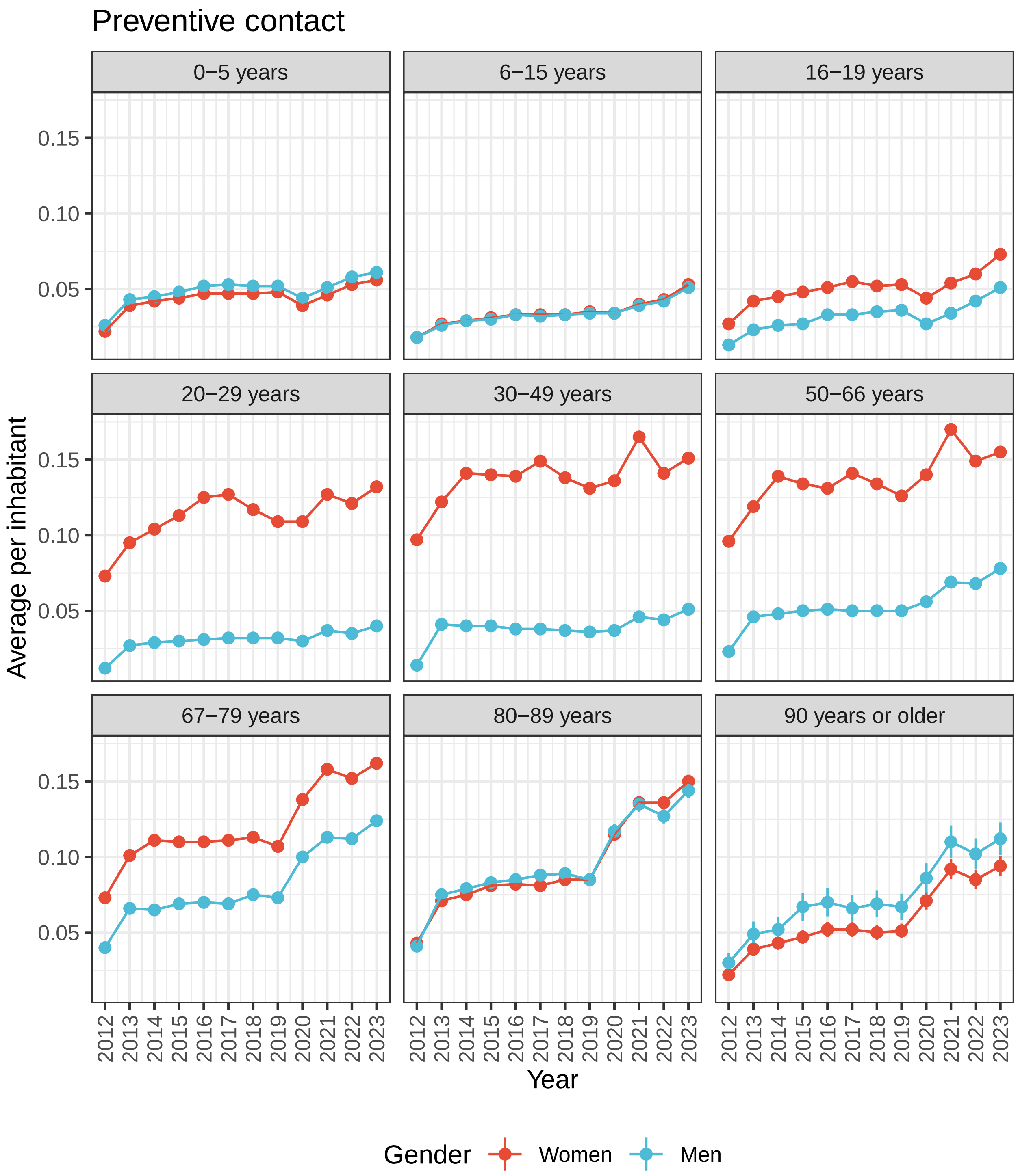

Preventive contact
0−5 years
6−15 years
16−19 years
20−29 years
30−49 years
50−66 years
67−79 years
80−89 years
90 years or older
0.15
0.10
0.05
Average per inhabitant
2012
2013
2014
2015
2016
2017
2018
2019
2020
2021
2022
2023
Year
Gender
Women
Men

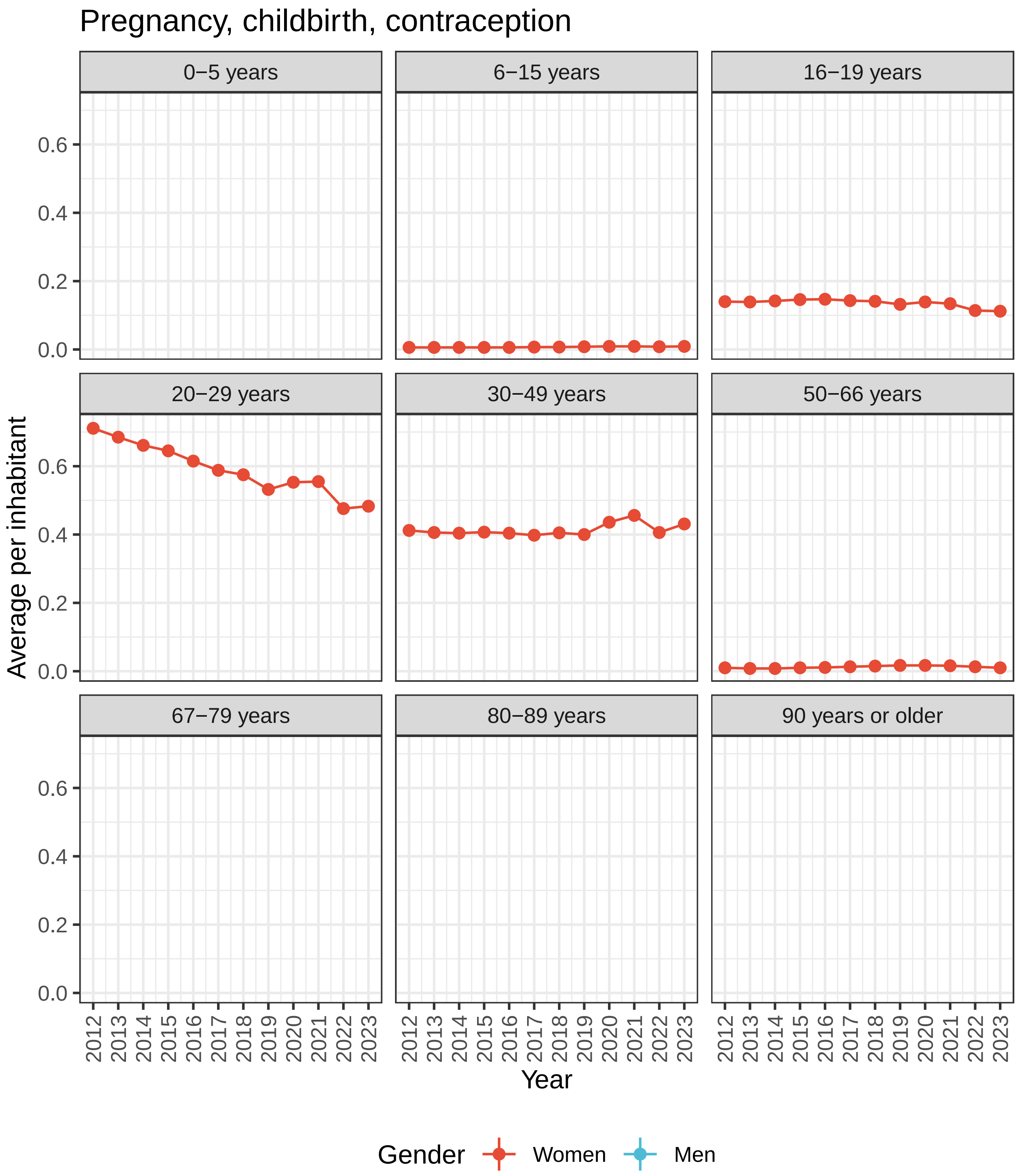

Pregnancy, childbirth, contraception
0−5 years
6−15 years
16−19 years
20−29 years
30−49 years
50−66 years
67−79 years
80−89 years
90 years or older
Average per inhabitant
0.0
0.2
0.4
0.6
2012
2013
2014
2015
2016
2017
2018
2019
2020
2021
2022
2023
Year
Gender
Women
Men

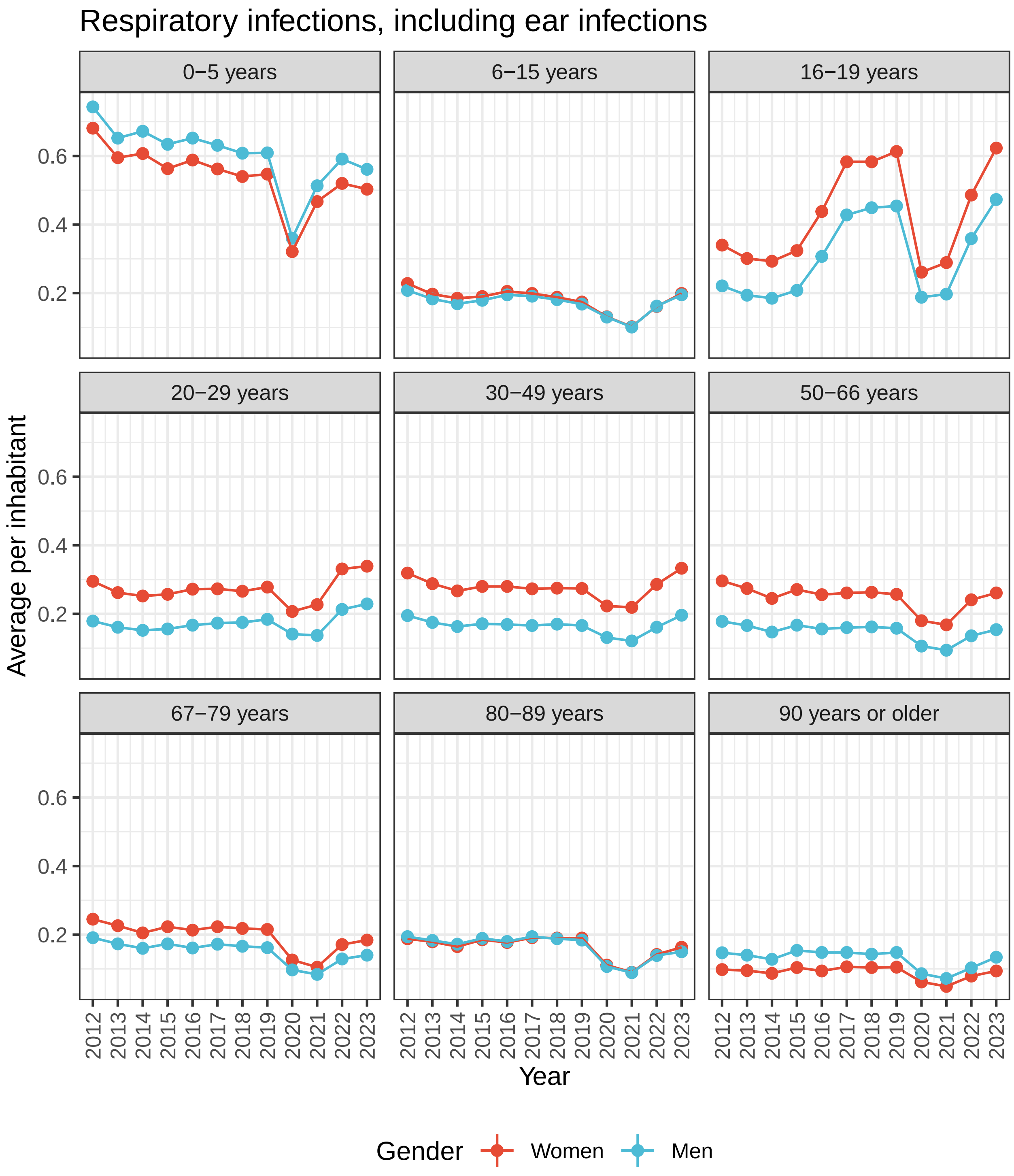
Respiratory infections, including ear infections
0−5 years
6−15 years
16−19 years
20−29 years
30−49 years
50−66 years
67−79 years
80−89 years
90 years or older
Average per inhabitant
0.2
0.4
0.6
2012
2013
2014
2015
2016
2017
2018
2019
2020
2021
2022
2023
Year
Gender
Women
Men

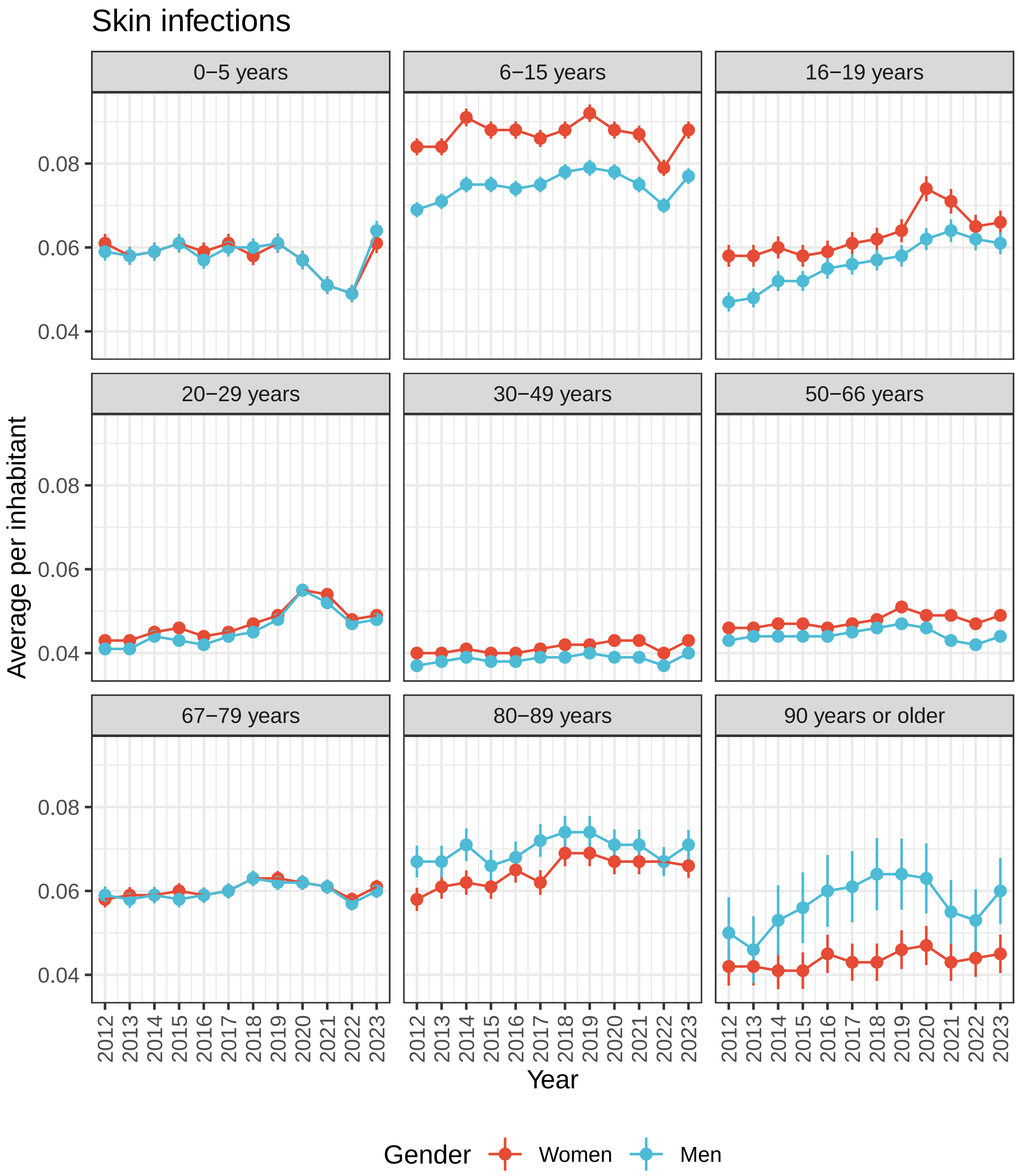
Skin infections
0−5 years
6−15 years
16−19 years
20−29 years
30−49 years
50−66 years
67−79 years
80−89 years
90 years or older
Average per inhabitant
0.04
0.06
0.08
Year
2012
2013
2014
2015
2016
2017
2018
2019
2020
2021
2022
2023
Gender
Women
Men
